# Transcriptional repression by FEZF2 restricts alternative identities of cortical projection neurons

**DOI:** 10.1016/j.celrep.2021.109269

**Published:** 2021-06-22

**Authors:** Jeremiah Tsyporin, David Tastad, Xiaokuang Ma, Antoine Nehme, Thomas Finn, Liora Huebner, Guoping Liu, Daisy Gallardo, Amr Makhamreh, Jacqueline M. Roberts, Solomon Katzman, Nenad Sestan, Susan K. McConnell, Zhengang Yang, Shenfeng Qiu, Bin Chen

**Affiliations:** 1 Department of Molecular, Cell, and Developmental Biology, University of California, Santa Cruz, 1156 High Street, Santa Cruz, CA 95064, USA; 2 Department of Basic Medical Sciences, University of Arizona College of Medicine – Phoenix, Phoenix, AZ 85004, USA; 3 State Key Laboratory of Medical Neurobiology and MOE Frontiers Center for Brain Science, Institute for Translational Brain Research, Institutes of Brain Science, Department of Neurology, Zhongshan Hospital, Fudan University, Shanghai 200032, China; 4 Genomics Institute, University of California, Santa Cruz, Santa Cruz, CA 95064, USA; 5 Department of Neuroscience, Yale School of Medicine, New Haven, CT 06520, USA; 6 Department of Biology, Stanford University, Stanford, CA 94305, USA; 7 These authors contributed equally; 8 Lead contact

## Abstract

Projection neuron subtype identities in the cerebral cortex are established by expressing pan-cortical and subtype-specific effector genes that execute terminal differentiation programs bestowing neurons with a glutamatergic neuron phenotype and subtype-specific morphology, physiology, and axonal projections. Whether pan-cortical glutamatergic and subtype-specific characteristics are regulated by the same genes or controlled by distinct programs remains largely unknown. Here, we show that FEZF2 functions as a transcriptional repressor, and it regulates subtype-specific identities of both corticothalamic and subcerebral neurons by selectively repressing expression of genes inappropriate for each neuronal subtype. We report that TLE4, specifically expressed in layer 6 corticothalamic neurons, is recruited by FEZF2 to inhibit layer 5 subcerebral neuronal genes. Together with previous studies, our results indicate that a cortical glutamatergic identity is specified by multiple parallel pathways active in progenitor cells, whereas projection neuron subtype-specific identity is achieved through selectively repressing genes associated with alternate identities in differentiating neurons.

## In brief

Layer 6 corticothalamic and layer 5 subcerebral projection neurons connect the cerebral cortex to the thalamus and brainstem and spinal cord, respectively. Tsyporin et al. report that the transcription factor FEZF2 specifies the development of these neuronal identities by repressing expression of genes associated with alternate projection neuron subtypes.

## Graphical Abstract

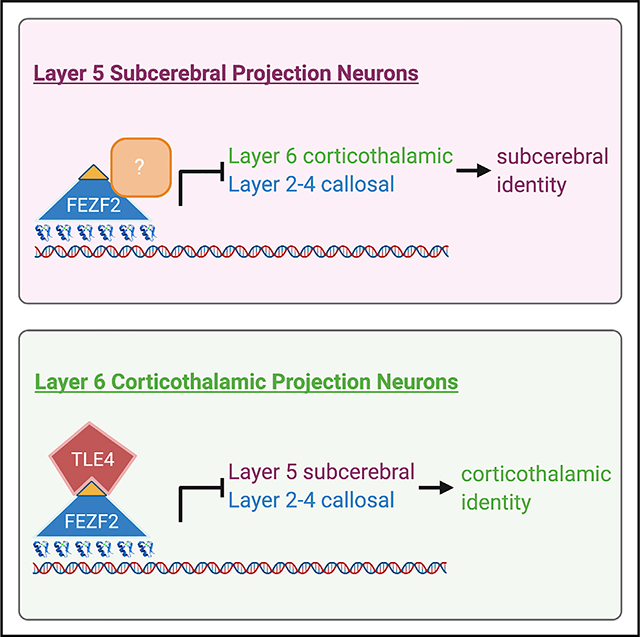

## INTRODUCTION

Projection neuron subtype identities in the developing cerebral cortex are established by expressing pan-cortical and subtype-specific genes, which execute terminal differentiation programs and bestow neurons with a glutamatergic phenotype and subtype-specific morphology, physiology, and axonal projections. Whether the pan-cortical glutamatergic phenotype and subtype-specific characteristics are regulated by the same genetic program or controlled by distinct genes remains largely unknown. In *C. elegans*, expression of terminal effector genes is activated by terminal selector genes, which are transcription factors that act in differentiating neurons by binding to common *cis*-regulatory elements in effector genes and activating their expression (Hobert and Kratsios, 2019). Whether similar mechanisms are utilized in developing mammalian brains is unknown, except the corticospinal motor neurons (CSMNs), a subset of subcerebral projection neurons specified by the transcriptional regulator FEZF2 (Lodato et al., 2014).

Although recent advances in single-cell RNA sequencing (scRNA-seq) technologies have allowed classification of neurons into different clusters based on gene expression in individual cells (Tasic et al., 2018), neocortical excitatory neurons can be broadly classified into 3 major subtypes based on where they project axons (Leone et al., 2008). Corticocortical neurons, located in layers 2–6, project axons to the ipsilateral (intracortical) or contralateral (callosal) cortex. The subcerebral neurons projecting to the thalamus (corticothalamic neurons) mostly reside in layer 6, whereas the subcerebral neurons projecting to the midbrain, hindbrain, and spinal cord are confined to layer 5B (O’Leary and Koester, 1993). Determining the molecular mechanisms underlying the differentiation of these neuronal subtypes is essential for understanding the regulatory logic of cell fate specification in the neocortex.

Prior studies have identified several genes that broadly specify the identities of cortical projection neuron subtypes and revealed that the development of these subtypes depends on a network of transcription factors that cross-inhibit one another’s expression. The zinc-finger transcription factor *Fezf2* is expressed in deep-layer neurons. It promotes a subcerebral neuronal identity and suppresses expression of subtype-determining genes for corticothalamic (*Tbr1*) and callosal (*Satb2*) neurons (Chen et al., 2005a, 2005b, 2008; Molyneaux et al., 2005). *Bcl11b*, also known as *Ctip2*, encodes a zinc-finger transcription factor expressed at high levels in layer 5 subcerebral neurons and at low levels in layer 6 corticothalamic neurons (McKenna et al., 2011). It regulates extension and fasciculation of subcerebral axons (Arlotta et al., 2005). *Tbr1* and *Sox5* are both expressed at high levels in corticothalamic projection neurons. They promote a corticothalamic neuronal fate and directly repress *Fezf2* expression and subcerebral identity in layer 6 neurons (Han et al., 2011; Kwan et al., 2008; Lai et al., 2008; McKenna et al., 2011). *Satb2* was initially reported to be specifically expressed in callosal neurons, where it promotes a callosal neuron identity by repressing genes, including *Bcl11b*, that are essential for subcerebral axon development (Alcamo et al., 2008; Britanova et al., 2008). Recent studies have shown that *Satb2* is also dynamically expressed in subcerebral neurons and is required for their fate specification (Leone et al., 2015; McKenna et al., 2015).

Despite the identification of these critical transcription factors, the molecular logic for cortical neuron subtype specification remains elusive. *Fezf2* has been the prototypic transcription factor for studying this process in subcerebral neurons. A recent study reported that *Fezf2* directly activates the expression of genes conferring glutamatergic and subcerebral neuronal identity and represses genes associated with GABAergic and callosal neuron phenotypes, suggesting that, similar to *C. elegans* neurons (Hobert and Kratsios, 2019), the subtype identities of cortical excitatory neurons are specified by terminal selector genes (Lodato et al., 2014). However, the ability of *Fezf2* to directly activate the expression of terminal effector genes has not been rigorously tested. *Fezf2* is expressed in both radial glial cells (RGCs) and in postmitotic neurons (Chen et al., 2005a, 2005b, 2008; Guo et al., 2013; Molyneaux et al., 2005), and it is unclear whether *Fezf2* is required in RGCs or in newly generated neurons to specify a subcerebral neuronal fate. The N-terminal of the FEZF2 protein contains an engrailed homology domain (EH1 domain), which is known to recruit the transducin-like enhancer of split (TLE) family transcriptional co-repressors (Hashimoto et al., 2000). It remains to be determined whether FEZF2 functions as a transcriptional repressor, an activator, or both during cortical development. Finally, previous studies and recent scRNA-seq analyses revealed that, in addition to subcerebral neurons, *Fezf2* is expressed in corticothalamic and deep-layer callosal neurons (Clare et al., 2017; Molyneaux et al., 2007; Tantirigama et al., 2016; Tasic et al., 2018). If *Fezf2* is the terminal selector gene for subcerebral neurons, what is the function of *Fezf2* in these other neuronal subtypes?

Members of the TLE family are co-repressors that are not capable of binding DNA, but instead interact with diverse sequence-specific DNA-binding transcription factors and repress transcription of downstream genes (Jennings and Ish-Horowicz, 2008; Turki-Judeh and Courey, 2012). TLE proteins critically regulate a wide range of organogenesis, including neurogenesis, osteogenesis, and hematopoiesis (Agarwal et al., 2015; Xing et al., 2018). One class of proteins that recruit TLEs are the homeodomain family of transcription factors, through the specific interaction between the EH1 motif of the homeodomain proteins and the WD40 repeats of the TLEs. This interaction is essential for NKX2.2, NKX2.9, NKX6.1, DBX2, and PAX7 to pattern the developing neural tube in mammals (Muhr et al., 2001). The presence of an EH1 domain in the N-terminal of FEZF2 suggests that it may recruit TLE4 as a transcriptional co-repressor. Indeed, a previous study demonstrated that FEZF2 and TLE4 proteins in *Xenopus* physically interact with each other (Zhang et al., 2014).

Here, we investigate how FEZF2 regulates cell fate specification of cortical projection neurons. We demonstrate that, instead of being a terminal selector gene, *Fezf2* functions as a selective repressor in multiple neuronal subtypes to repress the expression of genes associated with alternate subtypes. We show that, in corticothalamic neurons, FEZF2 and TLE4 co-regulate the molecular differentiation, dendritic morphology, and function of these neurons. Together with previous studies, our results suggest that distinct genetic programs act sequentially to regulate the differentiation of cortical projection neurons, with genes expressed in progenitor cells specifying the pan-cortical glutamatergic phenotype, and subtype-specifying transcription factors functioning in postmitotic cells to selectively repress the expression of genes associated with alternate subtype identities.

## RESULTS

### *Fezf2* is required in postmitotic neurons to regulate the development of both subcerebral and corticothalamic identities

**Figure 1.**
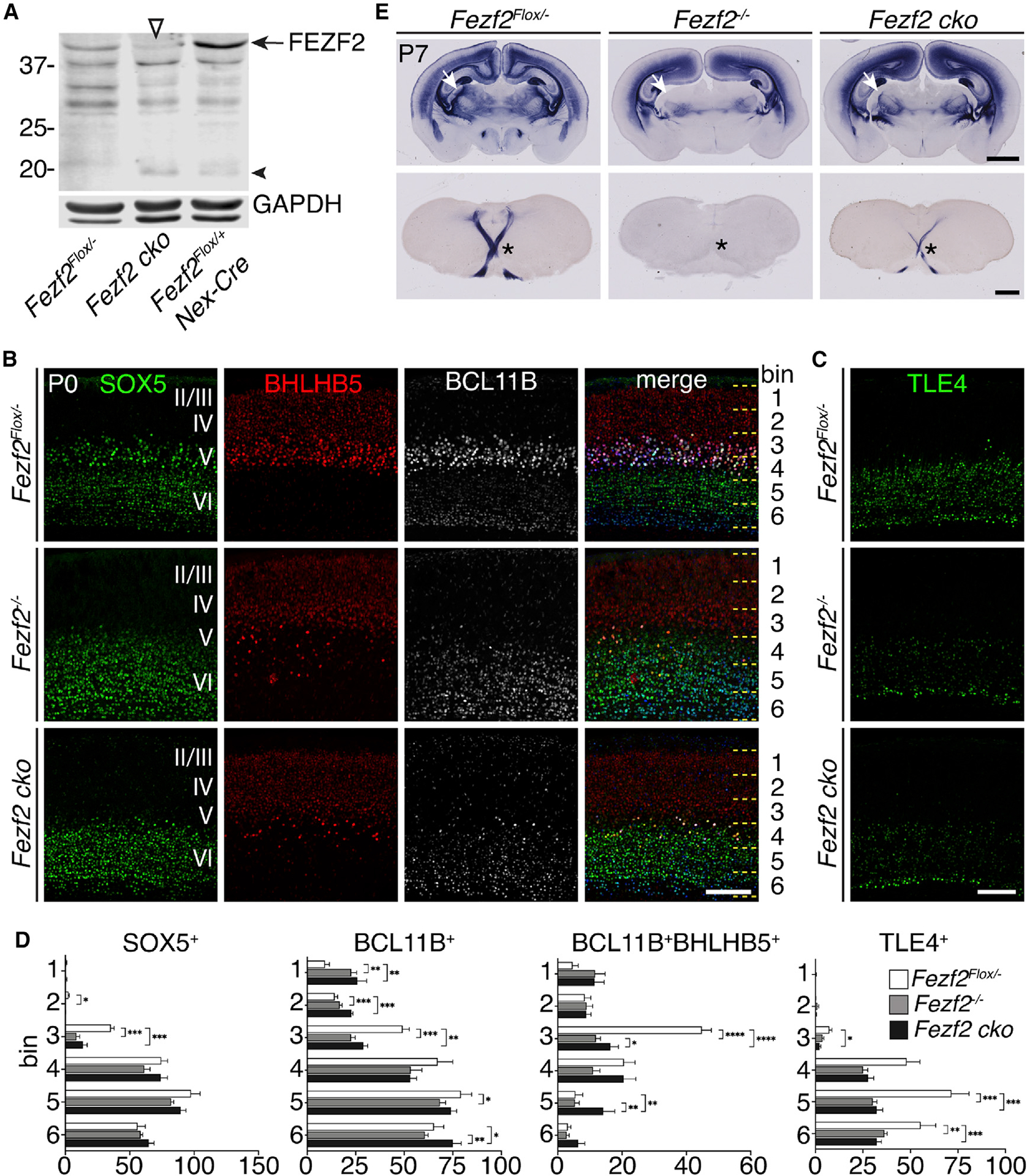
Fezf2 functions in postmitotic neurons to specify cell identity (A) Western blot showing a reduction of full-length FEZF2 protein (arrow) in the *Fezf2 cko* cortices. The empty triangle points to residual FEZF2 protein in the *Fezf2 cko* cortices. The recombined *Fezf2*^*Flox*^ allele produced a truncated FEZF2 (arrowhead) corresponding to the C-terminal half of the protein (see Figure S1A). (B and C) Immunostaining for SOX5, BHLHB5, BCL11B, and TLE4 on sections from P0 *Fezf2*^*Flox/*−^, *Fezf2*^−/−^, and *Fezf2 cko* mice. The cortices were divided into 6 equal bins, and the numbers of cells in each bin were counted. Scale bars: 100 μm. (D) Quantifications for marker^+^ neurons per 10,000 μm^2^ in each bin (width quantified: 250 μm). n = 3 brains per genotype, 3 sections per brain. In all graphs, error bars represent ± SEM. Statistical significance was determined using one-way ANOVA followed by post hoc Tukey’s t test (*p < 0.05; **p < 0.01; ***p < 0.001; ****p < 0.0001). (E) PLAP staining of brain sections of P7 *Fezf2*^*Flox/*−^, *Fezf2*^−/−^, and *Fezf2 cko* mice. The top row shows coronal cortical sections; the bottom row shows coronal sections at the level of pyramidal decussation. White arrows, dLGN; *pyramidal decussation. Scale bars: top row: 1 mm; bottom row: 500 μm. See also Figure S1.

**Figure 2.**
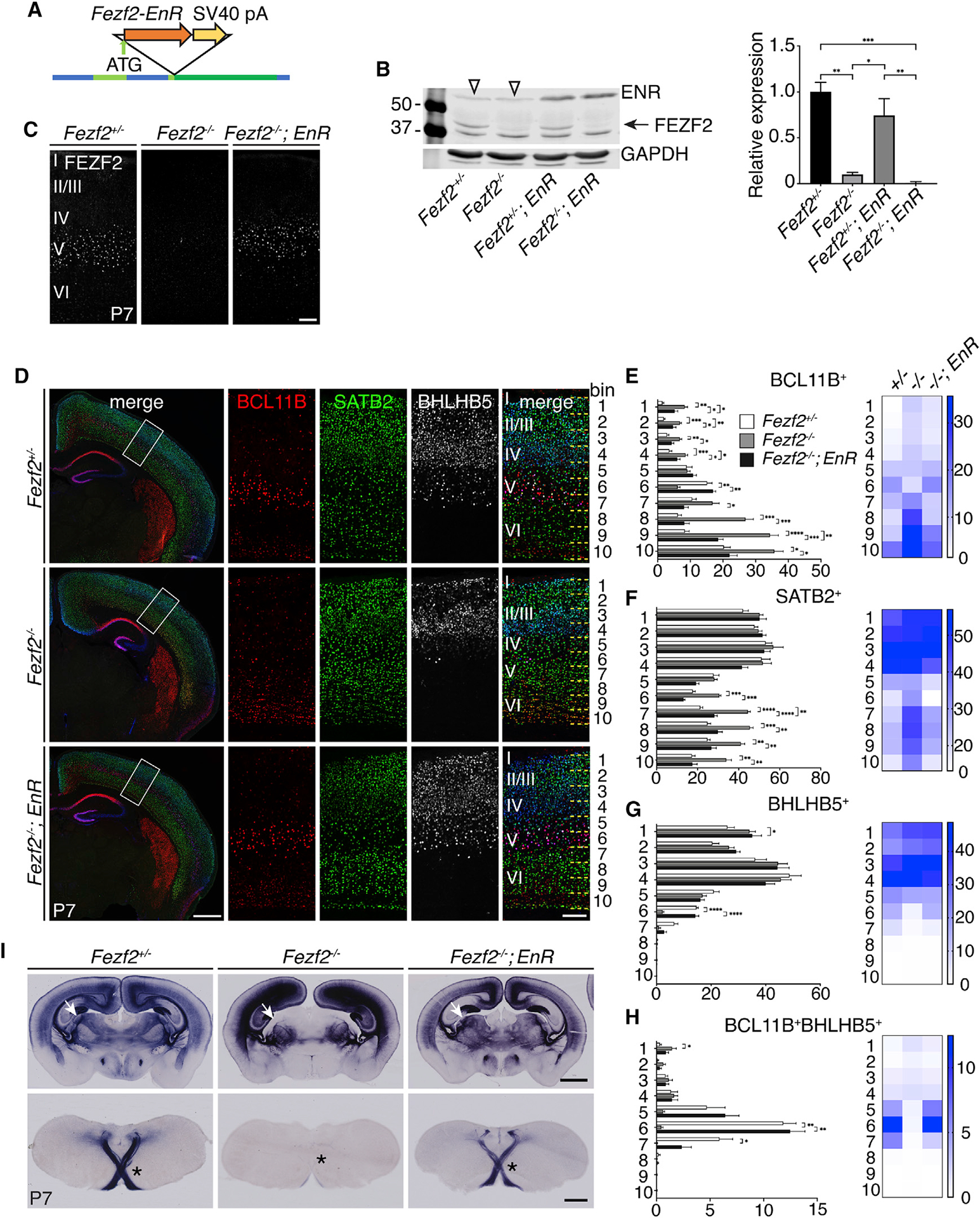
FEZF2 functions as a transcriptional repressor in cortical development (A) Strategy for generating the *Fezf2-EnR* BAC transgenic mouse line. (B) Western blot analysis of dissected cortices at P7. FEZF2 signal was normalized to GAPDH signal in each lane. n = 3 brains per genotype. Arrow, FEZF2 protein; empty triangles, non-specific bands. Signal intensities were measured using ImageStudioLite and normalized to a Gapdh internal loading control. Statistical significance was determined using one-way ANOVA followed by post hoc Tukey’s t test (*p < 0.05, **p < 0.01, ***p < 0.0001). Error bars represent SEM. (C) Immunostaining for FEZF2 on brain sections from P7 *Fezf2*^+/−^, *Fezf2*^−/−^, and *Fezf2*^−/−^; *Fezf2-EnR* (*EnR*) mice. Scale bar: 100 μm. (D) Immunostaining for BCL11B, SATB2, and BHLHB5 on P7 brain sections. Scale bar for low magnification: 500 μm. Scale bar for high magnification: 100 μm (E–H) Quantifications of marker^+^ cells per 10,000 μm^2^ in each bin. Heatmaps show the mean numbers of cells per 10,000 μm^2^ for each bin. n = 3 mice per genotype, 3 sections per brain. In all graphs, error bars represent ± SEM. Statistical significance was determined using one-way ANOVA followed by post hoc Tukey’s t test (*p < 0.05; **p < 0.01; ***p < 0.001; ****p < 0.0001). Binning was shown in (D). (I) PLAP staining of brain sections of P7 *Fezf2*^+/−^, *Fezf2*^−/−^, and *Fezf2*^−/−^*; EnR mice*. The top row shows coronal cortical sections; the bottom row shows coronal sections at the level of pyramidal decussation. Scale bars: 1 mm for top row; 500 μm for bottom row. White arrows, LGN; *pyramidal decussation. See also Figure S2.

To determine whether *Fezf2* acts in cortical RGCs or in postmitotic neurons to specify projection neuron subtype identities, we generated *Fezf2* conditional knockout mice (*Fezf2 cko*) using a *Fezf2*^*Flox*^ allele (Shim et al., 2012) and the *Nex*^*Cre*^ allele (Goebbels et al., 2006) to delete *Fezf2* in postmitotic neurons. Western blot analysis using a C-terminal FEZF2 antibody revealed that, although FEZF2 protein was absent in the *Fezf2*^−/−^ null mutant cortices, a faint band of full-length FEZF2 was detected in the *Nex*^*Cre*^ *Fezf2*^*Flox/*−^ (*Fezf2 cko*) cortices, indicating that the recombination was incomplete (Figures 1A and 2B). In addition, a smaller 20-kDa band was detected in *Fezf2 cko* cortices, corresponding to the truncated C-terminal half and DNA-binding domain of FEZF2 (Figures 1A, S1A, and S1B).

We compared cortices from *Fezf2 cko* mice to *Fezf2*^+/−^ and *Fezf2*^−/−^ cortices at postnatal day 0 (P0) and P7 (Figures 1B–1E and S1). The *Fezf2*^−^ null mutant allele contained a *PLAP* (human placenta alkaline phosphatase) knockin gene under the control of the endogenous *Fezf2* promoter and enabled us to directly observe the axons from *Fezf2*-expressing neurons (Chen et al., 2005a). The phenotypes of *Fezf2 cko* and *Fezf2*^−/−^ cortices were similar: (1) the expression of subcerebral neuronal genes, including BCL11B and BHLHB5, was significantly reduced in layer 5 neurons (Figures 1B and 1D); (2) expression of corticothalamic neuron genes, such as TBR1, and genes expressed at high levels in the callosal neurons, such as SATB2, was increased in layer 5 (Figures S1C and S1D), suggesting that subcerebral neurons adopted corticothalamic and callosal identities; (3) expression of corticothalamic neuronal genes, such as TLE4, ZFPM2 (FOG2), and FOXP2 was decreased in layer 6 neurons (Figures 1C, S1E, and S1F); (4) expression of BCL11B was increased in layer 6, demonstrating that the molecular distinction between subcerebral and corticothalamic neurons failed to be refined in these cells (Figures 1B–1D, S1B, S1E, and S1F); (5) PLAP^+^ subcerebral axons were significantly reduced in the pyramidal decussation (Figure 1E); and (6) consistent with a previous report (Diao et al., 2018), PLAP^+^ corticothalamic axons to the dorsal lateral geniculate nucleus (dLGN) and other thalamic nuclei were severely reduced in both *Fezf2 cko* and *Fezf2*^−/−^ mice (Figure 1E).

Despite these similarities, the phenotypes of *Fezf2 cko* and *Fezf2*^−/−^ cortices were not identical, likely due to incomplete recombination in *Fezf2 cko* mice (Figure 1A). Specifically, *Fezf2 cko* cortices contained a few BCL11B^+^BHLHB5^+^ subcerebral neurons (Figures 1B–1D), and some PLAP^+^ corticospinal axons were able to project to the pyramidal decussation (Figure 1E). However, the similarity between the phenotypes of *Fezf2 cko* and *Fezf2*^−/−^ mice indicates that *Fezf2* is required in postmitotic neurons to regulate the molecular identities and axonal projections of both subcerebral and corticothalamic neurons.

### FEZF2 functions as a transcriptional repressor to specify cortical projection neuron subtypes

FEZF2 consists of an N-terminal half containing an EH1 domain and other sequences and a C-terminal half consisting of six C2H2-type zinc-finger motifs (Hashimoto et al., 2000). Zinc-finger motifs are involved in DNA binding, and the EH1 domain recruits TLE family transcriptional co-repressors. To test whether FEFZ2 functions as a transcriptional repressor, an activator, or both, we generated expression plasmids encoding a full-length FEZF2 protein, a chimeric protein consisting of the transcriptional repressor domain of the engrailed protein (EnR) fused with the DNA-binding domain of FEZF2 (*pCAG-Fezf2-EnR*), or a chimeric protein consisting of the VP16 transcription activator domain (VP16) fused with the DNA-binding domain of FEZF2 (*pCAG-Fezf2-VP16*; Figure S2A). We co-electroporated each plasmid with a *pCAG-EGFP* plasmid into the cortical ventricular zone of embryonic day 15.5 (E15.5) wild-type embryos and examined the brains at P5 (Figure S2B). In all the electroporated brains, GFP^+^ neurons were located in layers 2 and 3 and GFP^+^ callosal axons were observed. GFP^+^ axons were not detectable in the thalamus or pons of brains electroporated with the *pCAG-EGFP* plasmid alone or in brains electroporated with *pFezf2-VP16* plasmids. However, both full-length FEZF2 and FEZF2-EnR directed layer 2 and 3 neurons to project GFP^+^ axons into the thalamus and cerebral peduncle (Figure S2B).

To determine whether FEZF2 functions primarily as a transcriptional repressor during deep-layer neuronal differentiation, we generated a transgenic line expressing the FEZF2-EnR chimeric protein using a bacterial artificial chromosome (BAC) (Figure 2A). This BAC consisted of a 200-kb region flanking the *Fezf2* gene. We inserted the FEZF2-EnR open reading frame at the endogenous *Fezf2* translation start site, immediately followed by a transcription termination signal. Western blot analysis confirmed that endogenous FEZF2 protein was not expressed from the *Fezf2-EnR* transgenic allele (Figure 2B). Immunostaining showed that expression of the FEZF2-EnR protein recapitulates that of endogenous *Fezf2* (Figure 2C).

We then determined whether FEZF2-EnR can rescue the defects resulting from a loss of *Fezf2* by comparing the brains of *Fezf2*^+/−^, *Fezf2*^−/−^, and *Fezf2*^−/−^; *Fezf2-EnR* mice (Figures 2D–2I and S2C–S2E). The expression patterns of subcerebral neuronal markers, such as BCL11B and BHLHB5 (Figures 2D, 2E, and 2G), were restored in layer 5 neurons in *Fezf2*^−/−^*;Fezf2-EnR* mice. Similarly, the expression patterns of corticothalamic neuronal genes, such as TLE4, FOXP2, and ZFPM2 (Figures S2C–S2E) were restored. The ectopic expression of TBR1 (data not shown), FOSL2 (Figure S2C), and SATB2 (Figures 2D and 2F) in layer 5 was no longer detected. Furthermore, PLAP^+^ axons projected into the pyramidal decussation, the dLGN, and other thalamic nuclei in the *Fezf2*^−/−^; *Fezf2-EnR* mice (Figure 2I). Thus, the *Fezf2-EnR* allele rescued the defects observed in layer 5 and layer 6 neurons in *Fezf2*^−/−^mice, indicating that FEZF2 functions as a transcriptional repressor.

### *Fezf2-EnR* broadly rescues gene expression defects in the *Fezf2*^−/−^ cortices

**Figure 3.**
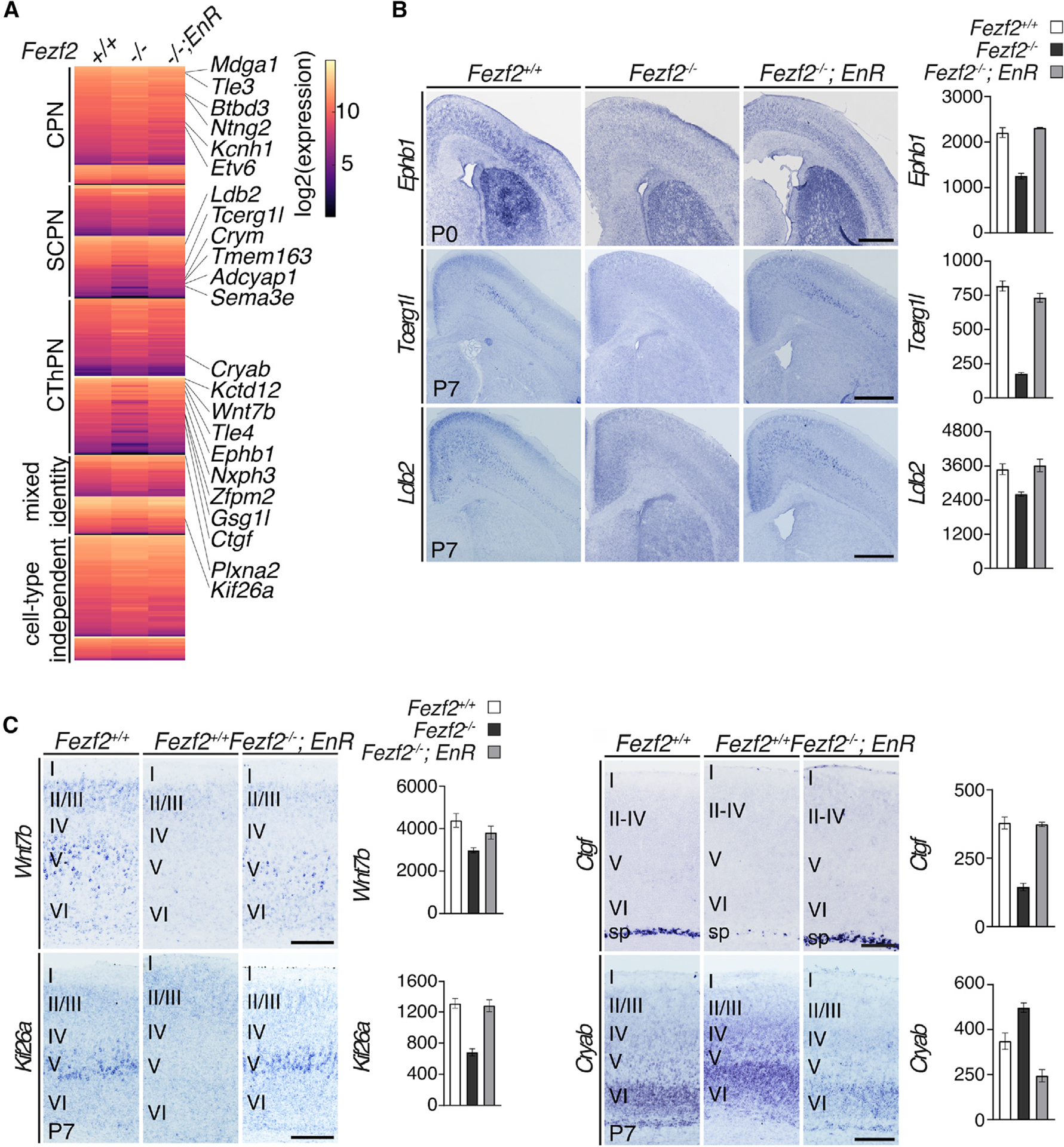
Genes mis-regulated in *Fezf2*^−/−^ cortices were enriched in projection neuron subtype-specific genes (A) Left heatmap shows normalized gene expression levels in P0 *Fezf2*^−/−^ (n = 4), *Fezf2*^−/−^; *Fezf2-EnR* (n = 2), and control *Fezf2*^+/+^ (n = 3) cortices. The subtype specificities for the mis-regulated genes were determined based on the DeCoN dataset. CPN, callosal projection neuronal genes; CThPN, corticothalamic neuronal genes; SCPN, layer 5 subcerebral projection neuronal genes; mixed identity, genes expressed in more than one neuronal subtype; cell-type independent, genes expressed in all subtypes. (B and C) *In situ* hybridization and RNA-seq analyses showed reduced expressions for *Ephb1*, *Tcerg1l*, *Ldb2*, *Wnt7b*, *Kif26a*, and *Ctgf* and increased expression of *Cryab* in *Fezf2*^−/−^ cortices, which were restored in the *Fezf2*^−/−^; *Fezf2-EnR* mice. Bar graphs showed normalized mRNA expression levels detected by RNA-seq in the cortices for the P0 *Fezf2*^+/+^ (n = 3 mice), *Fezf2* ^−/−^ (n = 4 mice), and *Fezf2*^−/−^; *Fezf2-EnR* (n = 2 mice) mice. Error bars represent SEM. Scale bars: 500 μm in (B) and 200 μm in (C).

To further investigate how FEZF2 regulates projection subtype identities, we performed bulk RNA-seq analysis of cortices from P0 *Fezf2*^−/−^, *Fezf2*^−/−^; *Fezf2-EnR*, and control *Fezf2*^+/+^ mice (Figures 3 and S3). Compared to the *Fezf2*^+/+^ mice, the expression levels of 408 genes were mis-regulated in the *Fezf2*^−/−^ cortices (p < 0.05). 140 genes showed reduced expression, and 268 showed increased expression. DAVID analysis (https://david.ncifcrf.gov) revealed that the top Gene Ontology (GO) terms associated with mis-regulated genes in *Fezf2*^−/−^ cortices were extracellular region, multicellular organism development, collagen fibril organization, synapse, and cell junction. We examined the expression of these mis-regulated genes in specific subtypes of cortical neurons using the DeCoN dataset (Molyneaux et al., 2015) and found that 88 of the 140 genes showing reduced expression in *Fezf2*^−/−^ cortices were subtype specific: 34 genes were enriched in subcerebral neurons; 43 in corticothalamic neurons; and 11 in corticocortical neurons. Among the 268 genes showing increased expression in *Fezf2*^−/−^ cortices, 125 were subtype specific: 54 were enriched in corticocortical neurons; 43 in corticothalamic neurons; and 28 in subcerebral neurons (Figure 3A). Consistent with the immunohistochemistry results (Figures 2 and S2), RNA-seq analysis of the *Fezf2*^−/−^; *Fezf2-EnR* cortices revealed that *Fezf2-EnR* broadly rescues these gene expression defects in the *Fezf2*^−/−^ cortices (Figure 3A).

We performed *in situ* hybridization to validate the RNA-seq analysis. Expressions of layer 5 subcerebral neuronal genes *Ephb1*, *Tcerg1*, and *Ldb2* were reduced in the *Fezf2*^−/−^ cortices, and their expressions were restored in layer 5 neurons in *Fezf2*^−/−^; *Fezf2-EnR* mice (Figure 3B). Corticothalamic neuronal gene *Wnt7b* and the subplate neuronal gene *Ctgf* showed reduced expressions in the *Fezf2*^−/−^ cortices; their expressions were rescued in the *Fezf2-EnR* mice (Figure 3C). *Kif26a*, a gene expressed in multiple neuronal subtypes, showed reduced expression in *Fezf2*^−/−^ cortices; its expression was restored in *Fezf2*^−/−^; *Fezf2-EnR* mice (Figure 3C). Expression of the corticothalamic neuronal gene, *Cryab*, was increased in the *Fezf2*^−/−^ cortices; its expression was restored in *Fezf2*^−/−^; *Fezf2-EnR* mice (Figure 3C). The broad rescue of gene expression defects in the *Fezf2*^−/−^ cortices by the *Fezf2-EnR* allele demonstrates that FEZF2 functions as a transcriptional repressor in specifying cortical projection neuron identities.

### TLE4 and FEZF2 are co-expressed in differentiating corticothalamic neurons and interact with each other

**Figure 4.**
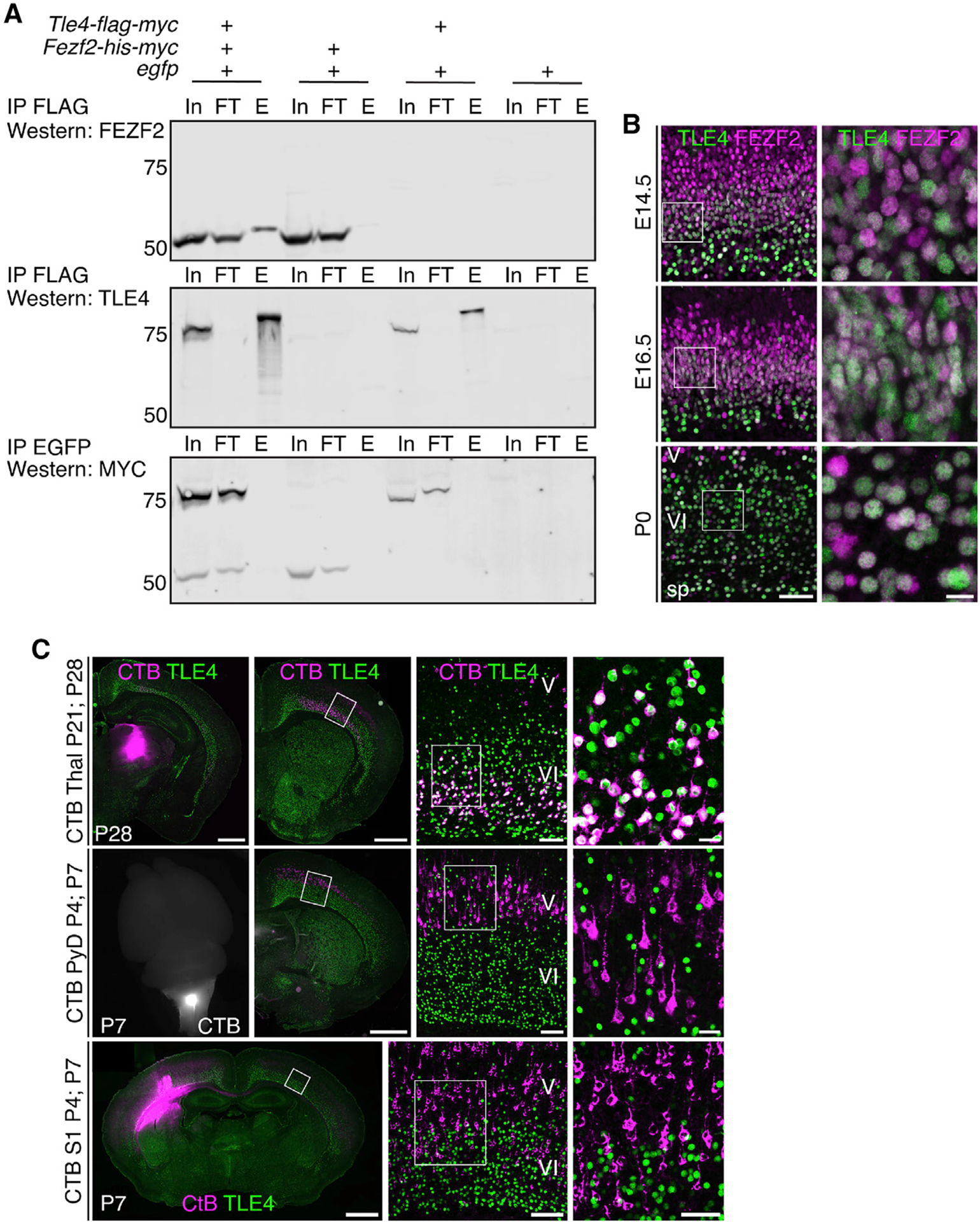
TLE4 and FEZF2 can bind to each other and are co-expressed in the corticothalamic neurons (A) Co-immunoprecipitation experiment showed that mouse FEZF2 and TLE4 proteins can bind each other. E, elution; FT, flow through; In, input. The bands around 50 KD are FEZF2-HIS-MYC; the bands around 75 KD are TLE4-FLAG-MYC. (B) Immunostaining for TLE4 and FEZF2 on brain sections from E14.5, E16.5, and P0 in wild-type mice. Low-mag single z-plane image scale bar: 50 μm; high-mag maximum z-projection image scale bar: 10 μm. (C) Combined retrograde tracing and immunostaining show that TLE4 is expressed in >99% of retrogradely labeled corticothalamic neurons in M1 (1,034 TLE4^+^CTB^+^ among 1,050 CTB^+^ cells), 0% of the traced subcerebral projection neurons (930 cells), and 0.1% of the callosal projection neurons (2 TLE4^+^CTB^+^ among 1,783 CTB^+^ cells). n = 3 mice for each injection location and 3 sections per brain were quantified. Low-mag scale bars: 1,000 μm. Scale bars in the second column from the right: 100 μm. Scale bars in the rightmost column: 20 μm.

The N-terminal region of the FEZF2 protein contains an EH1 motif, which can recruit the TLE family co-repressors (Muhr et al., 2001). A previous study reported that *Xenopus* FEZF2 and TLE4 proteins directly interact with each other (Zhang et al., 2014). Indeed, co-immunoprecipitation experiments revealed that the mouse FEZF2 and TLE4 also can bind to each other (Figure 4A). To determine whether FEZF2 recruits TLE4 to regulate the development of deep-layer neurons, we performed immunostaining using antibodies against FEZF2 and TLE4, which showed that the two proteins were co-expressed in layer 6 neurons (Figure 4B).

To identify the neuronal subtype that expresses TLE4, we performed retrograde tracing by injecting fluorescence-conjugated cholera toxin beta subunit (CTB) into the thalamus, pyramidal decussation, or contralateral cortex (Figure 4C). More than 99% of retrogradely labeled corticothalamic neurons expressed TLE4, whereas labeled subcerebral neurons and callosal neurons did not (<1%). Thus, TLE4 is specifically expressed in corticothalamic neurons.

### Corticothalamic axons developed normally in the *Tle4*^*LacZ/LacZ*^ mice

To test the function of *Tle4* in the specification and differentiation of corticothalamic neurons, we generated a *Tle4* mutant allele (*Tle4*^*LacZ*^) by inserting a *BGal-ires-Plap* cassette into the 4^th^ intron of the *Tle4* gene (Figure S4A). The BGAL and PLAP reporters enabled us to label the cell bodies and axons of *Tle4* heterozygous and mutant neurons (Figures S4B and S4D). In *Tle4*^+/*LacZ*^ brains, BGAL expression recapitulated the endogenous pattern of TLE4 expression (Figure S4B). Immunostaining revealed that TLE4 protein was not present in *Tle4*^*LacZ/LacZ*^ mice (Figure S4C). In both *Tle4*^+/*LacZ*^ and *Tle4*^*LacZ/LacZ*^ mice, PLAP^+^ axons extended from cortex to the thalamus, with no obvious difference between control and mutant brains (Figure S4D). We performed anterograde tracing by injecting adeno-associated virus (AAV)-mcherry virus into the primary motor cortex (M1), primary somatosensory cortex (S1), and primary visual cortex (V1) cortical areas, which confirmed that corticothalamic axons developed normally in *Tle4*^*LacZ/LacZ*^ mice (Figure S4E; data not shown).

### Molecular differentiation of corticothalamic neurons is defective in *Tle4*^*LacZ/LacZ*^ mice

**Figure 5.**
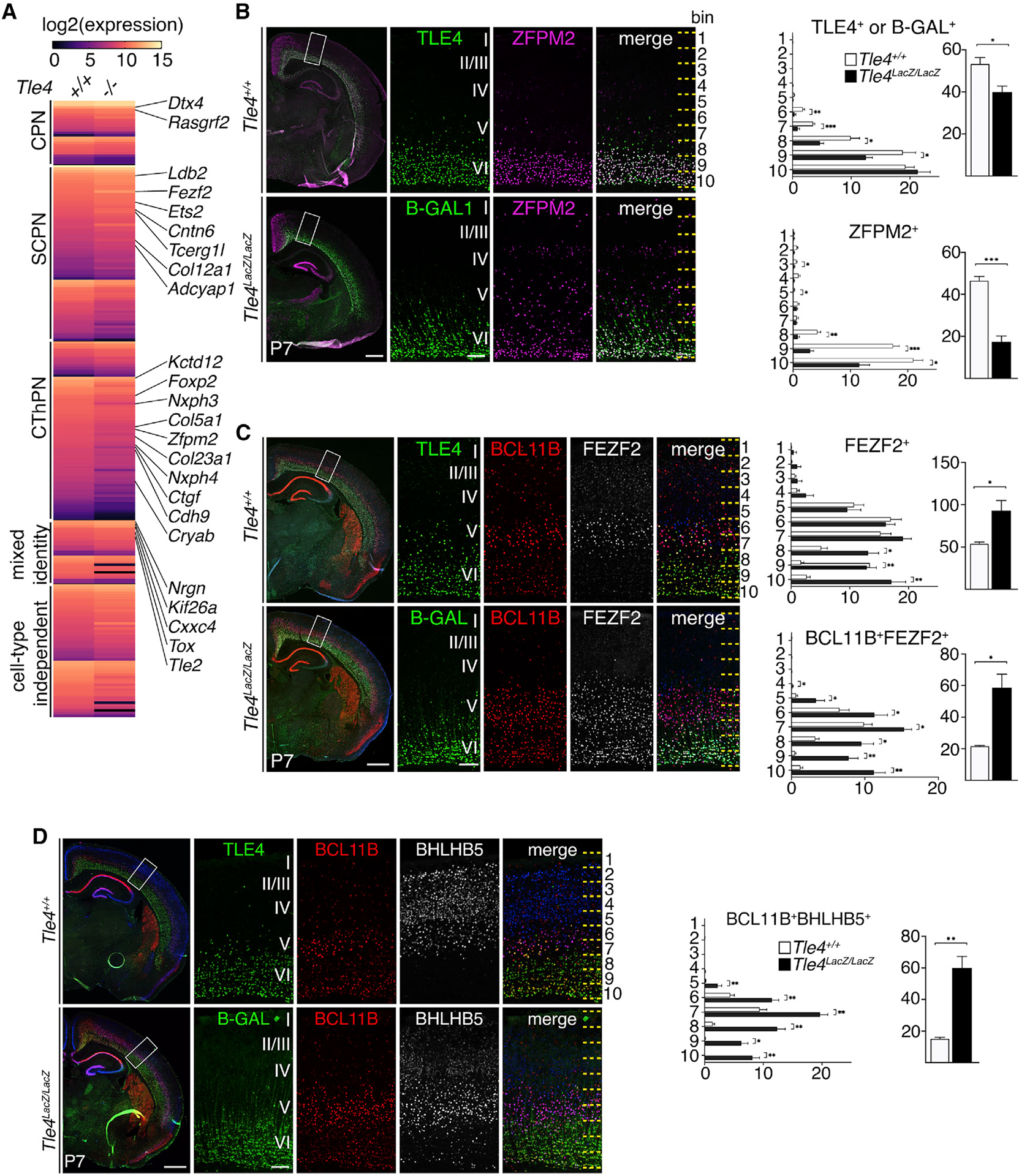
Molecular differentiation of corticothalamic neurons was defective in *Tle4^LacZ/LacZ^* brains (A) Heatmap showing normalized gene expression levels in the *Tle4*^+/+^ and *Tle4*^*LacZ/LacZ*^ cortices. The subtype specificities for the mis-regulated genes were determined based on the DeCoN dataset. (B) Immunostaining of TLE4 or B-GAL and ZFPM2 in the P7 brains and quantifications of the numbers of TLE4^+^ or B-GAL^+^ and ZFPM2^+^ cells by bin and total cell counts. (C) Immunostaining of TLE4 or B-GAL, BCL11B, and FEZF2 in the P7 brains and quantifications of the FEZF2^+^ and BCL11B^+^FEZF2^+^ cells by bin and total cell counts. (D) Immunostaining of TLE4 or B-GAL, BCL11B, and BHLHB5 in the P7 brains and quantifications of the BCL11B^+^BHLHB5^+^ cells n = 3 brains per genotype, 3 sections per brain. Quantifications of marker^+^ cells per 10,000 μm^2^ in each bin are shown. In all graphs, error bars represent ± SEM. Statistical significance was determined using the unpaired Student’s t test (*p < 0.05; **p < 0.01; ***p < 0.001). Scale bars: low-mag, 500 μm; high-mag, 100 μm. See also Figures S4–S6.

We performed RNA-seq analysis of control and *Tle4*^*LacZ/LacZ*^ P0 cortices (n = 3 mice for each genotype). 428 genes were mis-regulated in the *Tle4*^*LacZ/LacZ*^ cortices (p < 0.05; Student’s t test). Using the DeCoN dataset (Molyneaux et al., 2015), we examined the neuronal subtype-specific expression of all mis-regulated genes. Among the 228 genes with reduced expression, 11 were associated with corticocortical neurons, 56 were specifically expressed in corticothalamic neurons, and 24 in subcerebral neurons. Among the 200 upregulated genes, 14 were associated with corticocortical neurons, 45 were enriched in subcerebral neurons, and 14 in corticothalamic neurons (Figure 5A).

We performed immunohistochemistry and *in situ* hybridization to validate the RNA-seq results. Consistent with the normal corticothalamic axons observed in *Tle4*^*LacZ/LacZ*^ brains, the expression of TBR1 and SOX5, two genes essential for specifying a corticothalamic neuron identity, were not significantly affected (Figures S5A and S5B). However, the number of BGAL^+^ neurons was significantly reduced in layer 5 and layer 6a in *Tle4*^*LacZ/LacZ*^ mice (Figure 5B). Expression of other layer 6 neuron markers, such as ZFPM2 (Figure 5B) and FOXP2 (Figure S5C), was also significantly reduced. High expression levels of FEZF2, BCL11B, BHLHB5, *Ldb2*, and *Tcerg1l* are normally associated with layer 5 subcerebral neurons, but in *Tle4*^*LacZ/LacZ*^ mice, their expression was significantly increased in layer 6 (Figures 5C, 5D, S6A, and S6B).

The reduced numbers of BGAL^+^, ZFPM2^+^, and FOXP2^+^ cells in *Tle4*^*LacZ/LacZ*^ mice could be due to reduced neuronal production, increased cell death, or defective molecular differentiation, while the increased numbers of cells expressing BCL11B, FEZF2, and other subcerebral neuron markers in layer 6 could be due to a migration defect of layer 5 neurons or the mis-regulation of these genes in layer 6 neurons. To ascertain whether the production or migration of layer 5 and 6 neurons was affected in *Tle4*^*LacZ/LacZ*^ mice, we performed birthdating experiments by injecting EdU into pregnant mice on E12.5 or E13.5 and analyzing the brains of *Tle4*^*LacZ/LacZ*^ and littermate control mice at P7. For both labeling dates, we saw no significant change in the number or distribution of EdU^+^ cells in the deep layers of *Tle4*^*LacZ/LacZ*^ cortices (Figures S5A and S5B). We then stained sections from control and *Tle4*^*LacZ/LacZ*^ cortices at E14, P0, and P7 with an antibody against activated caspase 3 (AC3) and observed no significant difference in the numbers of AC3^+^ cells between genotypes at any age (Figure S6C).

Together, these results indicate that, in *Tle4*^*LacZ/LacZ*^ mice, corticothalamic neurons were generated in appropriate numbers, migrated to their normal laminar destinations, and projected axons to the thalamus. However, the molecular differentiation of these neurons was impaired, resulting in the expression of genes normally associated with layer 5 subcerebral neurons.

### Morphological and electrophysiological defects of corticothalamic neurons in *Tle4*^*LacZ/LacZ*^ mice

**Figure 6.**
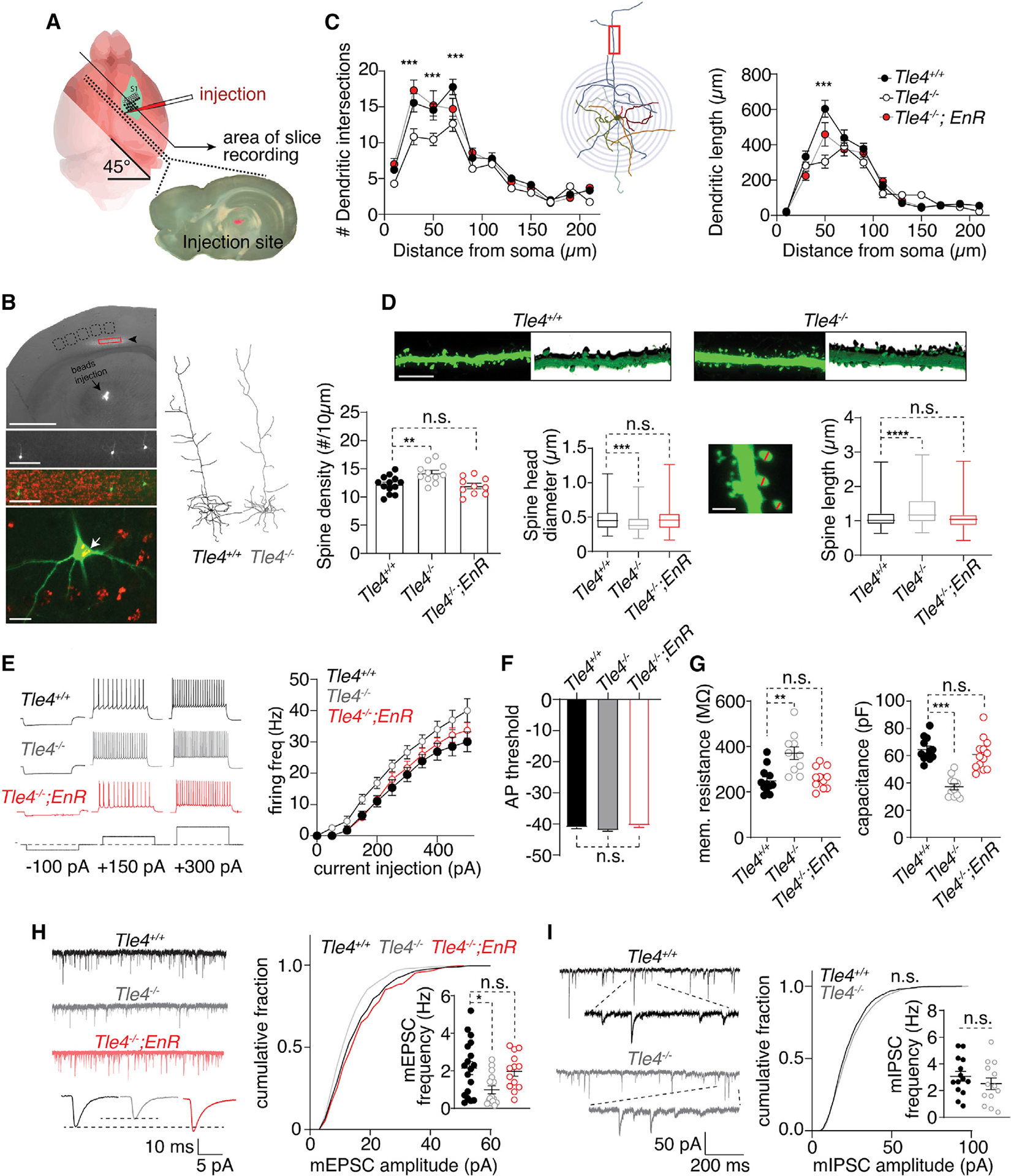
*Tle4^LacZ/LacZ^* mice show disrupted morphological and electrophysiological properties in corticothalamic neurons in S1, which were rescued by the *Fezf2-EnR* allele (A) Schematic illustration of retrobeads injection into VPM of thalamus and preparation of S1 slices for patch-clamp recording. (B) A brightfield image overlaid with bead^+^ layer 6 corticothalamic neurons (arrowhead). Scale bars: 1 mm top image; 100 μm middle images. An enlarged view shows a beads^+^ corticothalamic neuron and its morphology (white arrow) revealed by biocytin-avidin-Alexa 488. Scale bar: 20 μm. Right: two examples of representative dendritic arbors reconstructed from the beads^+^ corticothalamic neurons in a *Tle4*^+/+^ and a *Tle4*^*LacZ/LacZ*^ mouse. (C) Sholl analysis on the dendritic intersection numbers and dendritic length. Left panel shows a significant difference among the genotypes (F_(2,297)_ = 21.7; p < 0.0001. Tukey’s multiple comparison tests: between *Tle4*^+/+^ [n = 4 mice, 9 cells] and *Tle4*^*LacZ/LacZ*^ mice [n = 5 mice, 11 cells], p < 0.0001; between *Tle4*^+/+^ and *Tle4*^*LacZ/LacZ*^; *Fezf2-EnR* [n = 4 mice, 10 cells] mice, p = 0.96. ***p < 0.001, between *Tle4*^+/+^ and *Tle4*^*LacZ/LacZ*^ mice, Sidak’s multiple comparison test). Right panel shows dendritic length distribution. Genotype has a significant effect (F_(2,253)_ = 7.54; p = 0.0007. Tukey’s multiple comparisons test: between *Tle4*^+/+^ and *Tle4*^*LacZ/LacZ*^, p = 0.0006; between *Tle4*^+/+^ and *Tle4*^*LacZ/LacZ*^; *Fezf2-EnR*, p = 0.42. ****p < 0.0001, between *Tle4*^+/+^ and *Tle4*^*LacZ/LacZ*^, Sidak’s multiple comparison test). (D) Representative images of dendritic spines and their 3D projection images. Compared to the *Tle4*^+/+^ neurons (n = 4 mice, 12 cells, 20 spine lengths, 184 spine diameters), there was a significant increase in spine density (**p = 0.007; one way ANOVA with Dunnett’s multiple comparison) and spine length (****p < 0.0001; Kruskal-Wallis test followed by Dunn’s multiple comparison test) and a significant decrease in spine head diameter (**p < 0.0013) for the *Tle4*^*LacZ/LacZ*^ corticothalamic neurons (n = 4 mice, 11 cells, 209 spine lengths, 194 spine diameters). No significant difference in spine density, spine length, or spine head diameter was detected between the corticothalamic neurons in *Tle4*^+/+^ and *Tle4*^*LacZ/LacZ*^; *Fezf2-EnR* mice (n = 4 mice, 10 cells, 177 spine lengths, 192 spine diameters). Scale bars: 10 μm top image; 2 μm lower image. (E) Representative action potential firing responses from a *Tle4*^+/+^, *Tle4*^*LacZ/LacZ*^, and a *Tle4*^*LacZ/LacZ*^; *Fezf2-EnR* corticothalamic neuron. Genotype has a significant effect on the current-AP responses (F_(2, 165)_ = 24.1; p < 0.0001). Compared to *Tle4*^+/+^ neurons (n = 5 mice, 18 cells), *Tle4*^*LacZ/LacZ*^ neurons (n = 6 mice, 12 cells) show increased firing in response to depolarizing current steps (p < 0.0001; Dunnett’s multiple comparison), which was reversed in *Tle4*^*LacZ/LacZ*^; *Fezf2-EnR* mice (n = 5 mice, 13 cells; p = 0.14). (F) AP threshold did not differ between *Tle4*^+/+^ (n = 5 mice, 18 cells), *Tle4*^*LacZ/LacZ*^ (n = 6 mice, 12 cells), or *Tle4*^*LacZ/LacZ*^; *Fezf2-EnR* neurons (n = 5 mice, 13 cells; F_(2,32)_ = 2.5; p = 0.09; one-way ANOVA with Dunnett’s multiple comparison). (G) Genotype has a significant effect on membrane input resistance (F_(2,30)_ = 10.8; p = 0.0003; one-way ANOVA). *Tle4*^*LacZ/LacZ*^ neurons (n = 5 mice, 11 cells) show increased input resistance compared to *Tle4*^+/+^ neurons (n = 5 mice, 12 cells; p = 0.0003; Dunnett’s multiple comparison test), which was rescued in *Tle4*^*LacZ/LacZ*^; *Fezf2-EnR* (n = 4 mice, 11 cells) mice (p = 0.87). Genotype has a significant effect on membrane capacitance (F_(2,33)_ = 28.0; p < 0.0001). Compared to *Tle4*^+/+^ neurons (n = 5 mice, 12 cells), *Tle4*^*LacZ/LacZ*^ neurons (n = 5 mice, 11 cells) show decreased membrane capacitance (p = 0.0003; Dunnett’s multiple comparison test), which was rescued in *Tle4*^*LacZ/LacZ*^; *Fezf2-EnR* mice (n = 4 mice, 13 cells; between *Tle4*^+/+^ and *Tle4*^*LacZ/LacZ*^; *Fezf2-EnR*: p = 0.52). (H) Left: representative mEPSC traces from *Tle4*^+/+^, *Tle4*^*LacZ/LacZ*^, and *Tle4*^*LacZ/LacZ*^; *Fezf2-EnR* neurons. Right: cumulative plot on mEPSC amplitude is shown. Between *Tle4*^+/+^ (n = 5 mice, 8 cells, 1,135 measurements) and *Tle4*^*LacZ/LacZ*^ (n = 5 mice, 6 cells, 1,069 measurements), *p < 0.02, Kolmogorov-Smirnov test; between *Tle4*^*LacZ/LacZ*^ and *Tle4*^*LacZ/LacZ*^; *Fezf2-*EnR (n = 4 mice, 9 cells, 1,158 measurements), p = 0.57. Inset: a significant decrease in mEPSC frequency in *Tle4*^*LacZ/LacZ*^ neurons (n = 5 mice, 18 cells) between *Tle4*^+/+^ (n = 5 mice, 15 cells; *p = 0.015; Dunn’s multiple comparison following Kruskal-Wallis test) was restored by the *Fezf2-EnR* allele (n = 4 mice, 13 cells; between *Tle4*^+/+^ and *Tle4*^*LacZ/LacZ*^; *Fezf2-EnR*: p = 0.99; Dunn’s multiple comparison test). (I) No significant change in mIPSC amplitude and frequency was observed between *Tle4*^+/+^ (n = 5 mice, 14 cells) and *Tle4*^*LacZ/LacZ*^ neurons (n = 5 mice, 13 cells; cumulative amplitude: p = 0.43; frequency: p = 0.17. Kolmogorov-Smirnov test). Note that the *Tle4*^*LacZ*^ allele was labeled as *Tle4*^−^ in the figure to prevent crowding. All error bars represent ± SEM.

We next investigated whether *Tle4* is required for the morphological and functional differentiation of corticothalamic neurons. We injected retrobeads into the ventral posteromedial nucleus (VPM) of the thalamus (Landisman and Connors, 2007) of *Tle4*^+/+^ and *Tle4*^*LacZ/LacZ*^ mice (P27–P35) and performed whole-cell patch-clamp recordings and morphological analyses on labeled corticothalamic neurons in S1 (Figures 6A and 6B). Sholl analysis on reconstructed dendritic arbors revealed a significant reduction in the branching (F_(2,286)_ = 3.4; p = 0.034) and length (F_(2,253)_ = 7.3; p = 0.0009) of dendrites in mutant mice (Figure 6C). Mutant corticothalamic neurons also show significantly increased spine density, decreased spine head diameter, and increased spine length (Figure 6D).

Patch-clamp recordings revealed that corticothalamic neurons in *Tle4*^*LacZ/LacZ*^ mice exhibited increased excitability, as demonstrated by an increased number of action potentials (APs) firing in response to current steps (Figure 6E; F_(1,110)_ = 38.9; p < 0.001); however, the AP threshold was unaffected (Figure 6F). Mutant corticothalamic neurons showed an increased membrane resistance and decreased membrane capacitance (Figure 6G; p < 0.05 for both). The amplitude (p = 0.02; Kolmogorov-Smirnov test) and frequency (t_31_ = 2.7; p = 0.011) of miniature excitatory postsynaptic currents (mEPSC) were reduced in *Tle4*^*LacZ/LacZ*^ mice compared to wild-type mice (Figure 6H), but no significant change in the amplitude or frequency of miniature inhibitory postsynaptic currents (mIPSC) was observed (Figure 6I). These results collectively indicate that *Tle4* is critical for the morphological development and function of corticothalamic neurons in somatosensory cortex.

### Expression of FEZF2-EnR rescued the molecular, morphological, and functional defects of layer 6 neurons in *Tle4*^*LacZ/LacZ*^ cortices

**Figure 7.**
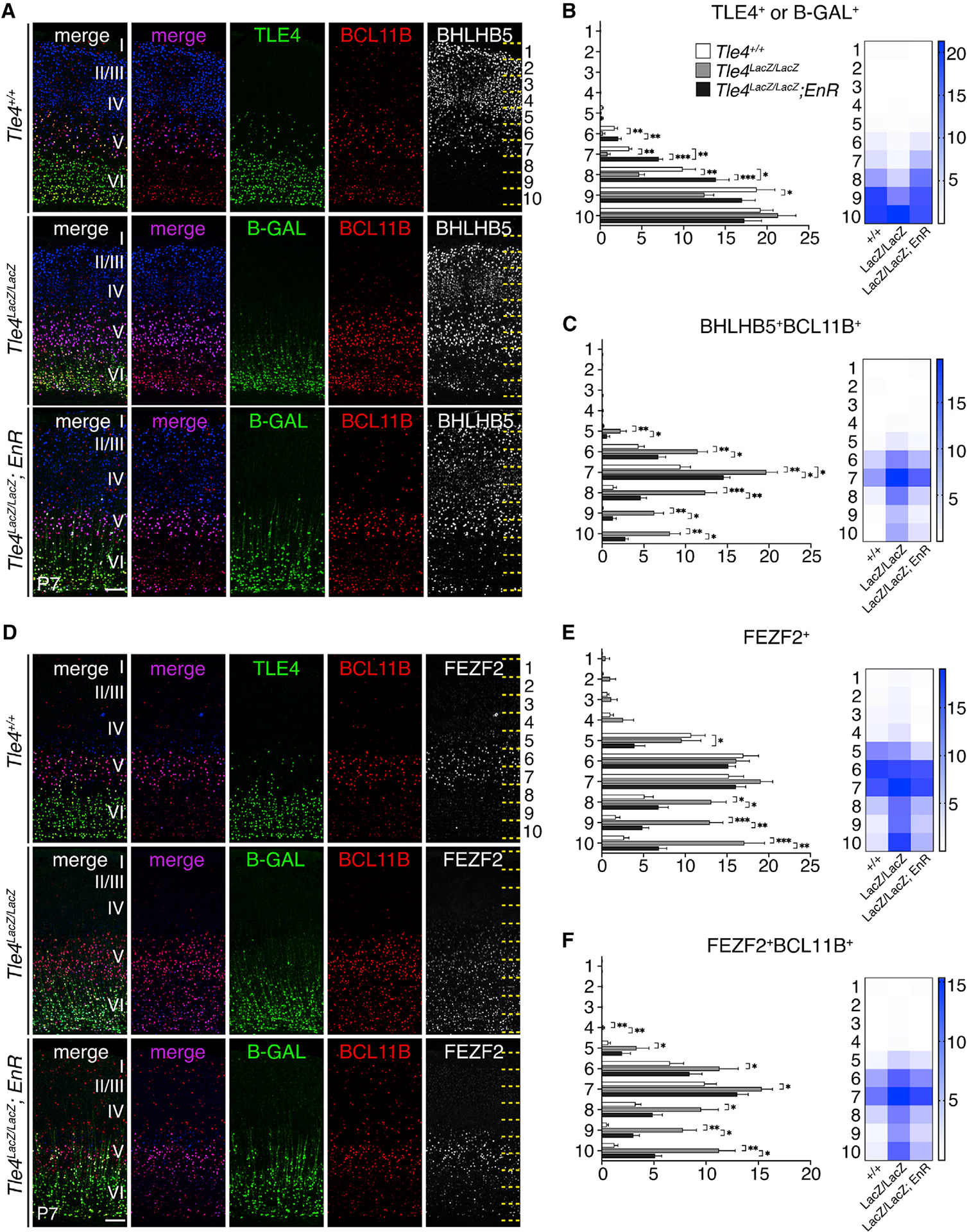
FEZF2-EnR represses the increased expression of subcerebral neuronal genes in the corticothalamic neurons in the *Tle4^LacZ/LacZ^* mice (A) Immunostaining of TLE4 or B-GAL, BCL11B, and BHLHB5 in the cortices of P7 *Tle4*^+/+^, *Tle4*^*LacZ/LacZ*^, and *Tle4*^*LacZ/LacZ*^; *Fezf2-EnR* mice. Scale bar: 100 μm. (B) Quantifications of TLE4^+^ cells in the *Tle4*^+/+^ cortices and the B-GAL^+^ cells in *Tle4*^*LacZ/LacZ*^ and *Tle4*^*LacZ/LacZ*^; *Fezf2-EnR* cortices. (C) Quantifications of the numbers of BCL11B^+^BHLHB5^+^ cells. (D) Immunostaining of TLE4, B-GAL, BCL11B, and FEZF2 in the cortices of P7 *Tle4*^+/+^, *Tle4*^*LacZ/LacZ*^, and *Tle4*^*LacZ/LacZ*^; *Fezf2-EnR* mice. Scale bar: 100 μm. (E) Quantifications of the FEZF2^+^ cells by bin. (F) Quantifications of the numbers of FEZF2^+^BCL11B^+^ cells. n = 3 brains per genotype, 3 sections per brain. In all graphs, error bars represent ± SEM. Statistical significance was determined using one-way ANOVA followed by post hoc Tukey’s t test (*p < 0.05; **p < 0.01; ***p < 0.001). See also Figure S6.

The co-expression of FEZF2 and TLE4 in corticothalamic neurons suggests that they function together in regulating the development of these neurons. To test this, we generated *Tle4*^*LacZ/LacZ*^; *Fezf2-EnR* mice and compared them to *Tle4*^+/+^ and *Tle4*^*LacZ/LacZ*^ mice. The number of B-GAL^+^ cells in *Tle4*^*LacZ/LacZ*^; *Fezf2-EnR* mice was restored to the number of TLE4^+^ cells seen in *Tle4*^+/+^ mice(Figures 7A and 7B). The expression of genes normally enriched in subcerebral neurons, such as BCL11B, BHLHB5, *Tcerg1l*, and *Ldb2*, was no longer increased in layer 6 neurons in *Tle4*^*LacZ/LacZ*^; *Fezf2-EnR* mice (Figures 7A, 7C–7F, S6A, and S6B). However, expression of corticothalamic neuron genes, including ZFPM2 and FOXP2, and the subplate gene *Ctgf*, was not restored (Figures S6C–S6F; data not shown). These results show that FEZF2 and TLE4 function together to prevent the high-level expression of subcerebral neuronal genes in corticothalamic neurons. They also suggest that, besides serving as a transcriptional co-repressor for FEZF2, TLE4 has additional functions in regulating the molecular differentiation of corticothalamic neurons.

We next compared the dendritic morphology and function of corticothalamic neurons in *Tle4*^*LacZ/LacZ*^; *Fezf2-EnR* mice to *Tle4*^+/+^ mice (Figure 6). Sholl analysis revealed that *Fezf2-EnR* rescued the decreased dendritic branching (p = 0.96) and length (p = 0.42) observed in *Tle4*^*LacZ/LacZ*^ corticothalamic neurons (Figure 6C). The changes in spine density (p = 0.88), spine head diameter (p = 0.98), and spine length (p = 0.99) were also reversed (Figure 6D). Patch-clamp experiments showed that the *Fezf2-EnR* allele normalized the increased neuronal excitability associated with *Tle4*^*LacZ/LacZ*^ neurons (Figure 6E). Corticothalamic neurons in *Tle4*^*LacZ/LacZ*^; *Fezf2-EnR* and wild-type mice showed similar membrane resistance (p = 0.53), capacitance (p = 0.87; Figure 6G), and mEPSC amplitude cumulative distribution and frequency (Figure 6H). These data suggest that FEZF2 and TLE4 together regulate the morphological and functional differentiation of corticothalamic projection neurons.

## DISCUSSION

Deciphering the molecular logic for establishing neuronal subtype identities in the developing cerebral cortex is fundamental for understanding how neuronal diversity is established in the mammalian brain. In this study, we examined the molecular control of two broad classes of cortical projection neurons—the corticothalamic and the layer 5 subcerebral projection neurons—by focusing on the function of FEFZ2.

Prior studies demonstrated that FEFZ2 is a master regulator for CSMNs, a subpopulation of layer 5 subcerebral neurons. In *Fezf2*^−/−^ mice, layer 5 subcerebral neurons were absent, and instead, the mutant layer 5b neurons demonstrated the molecular features and axonal projection patterns of corticothalamic or corticocortical neuron subtypes (Chen et al., 2005a, 2008; Molyneaux et al., 2005). Complementing these studies, ectopic expression of *Fezf2* in late cortical progenitors or upper-layer neurons or in the progenitors of the lateral ganglionic eminence led to the differentiation of neurons with gene expression and axon projections associated with corticothalamic or subcerebral neurons (Chen et al., 2005b, 2008; De la Rossa et al., 2013; Lodato et al., 2014; Molyneaux et al., 2005; Rouaux and Arlotta, 2010, 2013), suggesting that FEZF2 functions as a selector gene for subcerebral neurons. To test this, Lodato et al. (2014) performed gene expression analyses of cortical progenitors and neurons that overexpressed FEZF2 and chromatin immunoprecipitation sequencing (ChIP-seq) analysis of neurospheres that overexpressed FEZF2-FLAG. Results from these experiments led the authors to conclude that FEZF2 functions as both a transcriptional activator and a repressor. They suggested that FEZF2 directly induces CSMN glutamatergic identity by activating expression of genes including *Vglut1* (*Slc17a7*) and that it inhibits a GABAergic fate by repressing the transcription of genes such as *Gad1* (Lodato et al., 2014). They further reported that FEZF2 directly activates CSMN-specific genes by binding to their proximal promoters and represses genes expressed in corticocortical projection neurons (Lodato et al., 2014).

Here, we directly tested whether FEZF2 functions as a transcriptional activator, a repressor, or both by comparing the activities of full-length FEZF2, FEZF2-EnR, and FEZF2-VP16 chimeric proteins using two different assays. Results from both overexpression and rescue experiments showed that FEZF2-EnR recapitulated the activity of full-length FEZF2 protein, while the FEZF2-VP16 was less relevant in our assays. Thus, in contrast to prior work, our studies demonstrate that FEZF2 functions primarily as a transcriptional repressor, in context of the formation of the corticospinal tract and neuronal identity. It is not clear why our results differ so markedly from those of Lodato et al. (2014), although it seems possible that ChIP-seq experiments may have overestimated the binding sites for FEZF2 in normal cortical neurons (Jain et al., 2015; Teytelman et al., 2013).

FEZF2 is required for establishing the molecular identity and axonal projections of both subcerebral and corticothalamic neurons. In the absence of *Fezf2*, subcerebral neurons exhibit molecular features and axonal projection patterns associated with corticothalamic or corticocortical neuron subtypes (Chen et al., 2005a, 2008; McKenna et al., 2011). Previous reports (Diao et al., 2018; Komuta et al., 2007) and our current study show that, in *Fezf2*^−/−^ mice, corticothalamic neurons also exhibit defects in gene expression and axonal projections. We found that *Fezf2 cko* corticothalamic neurons showed increased expression of certain subcerebral neuronal genes, such as BCL11B and the truncated FEZF2, indicating that corticothalamic neurons partially assume the molecular identity of subcerebral neurons in the absence of *Fezf2* function. Together, these results suggest that FEZF2 inhibits the expression of distinct and specific target genes in subcerebral neurons and in corticothalamic neurons, and by doing so, FEZF2 prevents each class of neurons from adopting an alternate neuronal subtype identity. Consistent with this, the *Fezf2-EnR* allele rescued the molecular identities and axonal projections of both the subcerebral and corticothalamic subtypes in *Fezf2*^−/−^ mice.

How does FEZF2 function as a transcriptional repressor? One possibility is that FEZF2 binds to an enhancer or promoter sequence and physically prevents the binding of a transcriptional activator. Another possibility is that the binding of FEZF2 to an enhancer or promoter recruits additional transcriptional co-repressor(s), and together they actively repress gene expression. In *Nex-Cre; Fezf2*^*Flox/*−^ mice, a truncated FEZF2 protein consisting of just the DNA binding domain was expressed, yet in these mice, subcerebral and corticothalamic neurons and their axons showed similar defects as in *Fezf2*^−/−^ null mutant mice (Figures 1 and S1). This result indicates that the N-terminal half of the FEZF2 protein is essential for its transcriptional repressor function, likely by recruiting transcriptional co-repressors, and that FEZF2 is unlikely to repress gene expression simply by blocking the binding of a transcriptional activator.

Indeed, FEZF2 contains an EH1 motif, which can recruit TLE family transcription co-repressors (Hashimoto et al., 2000). The co-expression of FEZF2 and TLE4 in corticothalamic neurons suggests that TLE4 may be one of its co-repressors. Similar to *Fezf2*^−/−^ mice, corticothalamic neurons in *Tle4*^*LacZ/LacZ*^ mice showed increased expression of genes associated with subcerebral neurons, indicating that TLE4 is involved in refining the molecular identity of corticothalamic neurons by preventing high-level expression of some subcerebral neuronal genes. We found that the *FEZF2-EnR* allele, which does not depend on TLE family transcription co-repressors for its function, prevented the high expression levels of subcerebral neuronal genes and restored the functional properties of corticothalamic neurons in *Tle4*^*LacZ/LacZ*^ mice. These results support the conclusion that FEZF2 and TLE4 together repress the expression of FEZF2, BCL11B, and BHLHB5 in corticothalamic neurons. However, the reduced expression of corticothalamic neuronal genes, such as FOG2 and FOXP2 in *Tle4*^*LacZ/LacZ*^ mice, was not rescued by the *Fezf2-EnR* allele. Furthermore, although corticothalamic axons to the dLGN were missing in *Fezf2*^−/−^ mice, they were present in *Tle4*^*LacZ/LacZ*^ mice. Thus, although FEZF2 and TLE4 together refine the molecular differentiation and function of corticothalamic neurons, each plays additional independent roles. The identity of possible co-repressor(s) for FEZF2 in subcerebral neurons remains unknown.

Recent progress and our current study show that the subtype identity of a cortical projection neuron is specified in the postmitotic stage. Multiple transcription factors, including *Tbr1* (Han et al., 2011; McKenna et al., 2011), *Sox5* (Kwan et al., 2008; Lai et al., 2008), *Fezf2* (Chen et al., 2005a, 2005b, 2008; Molyneaux et al., 2005), and the chromatin remodeling protein *Satb2* (Alcamo et al., 2008; Britanova et al., 2008; Leone et al., 2015; McKenna et al., 2015), are essential for specifying cortical projection neuron subtype identities. A common phenotype of mice with mutations in these genes is that the affected neuronal subtypes exhibit gene expression profiles and axonal projection patterns associated with alternate neuronal subtypes. This suggests that TBR1, SOX5, and SATB2 likely also function as selective repressors in their respective neuronal subtypes to inhibit expression of genes associated with alternate identities. In the future, it will be important to test whether these proteins act as transcriptional repressors, activators, or both in the context of cortical projection neuron subtype specification. Another common feature shared by the *Tbr1*, *Sox5*, and *Satb2* genes is that all are expressed in postmitotic neurons. Although *Fezf2* is expressed by both cortical RGCs and deep-layer neurons, our results indicate that it is required in postmitotic neurons for specifying cortical neuron subtype identities. These results suggest that projection neuron subtype-specific features are established through repressing genes associated with alternate subtype identities during postmitotic neuronal differentiation.

Although different subtypes of cortical projection neurons have distinct morphologies, axonal projection patterns, and molecular profiles, they share a common cortical regional identity and use glutamate as an excitatory neurotransmitter. A fundamental question in brain development is whether a single genetic program specifies both the pan-cortical excitatory neuron identity and the subtype-specific identity of a cortical projection neuron or these features are specified by distinct genetic programs. Projection neuron subtype identities are mis-specified in *Fezf2*^−/−^, *Tbr1*^−/−^, *Sox5*^−/−^, and *Satb2*^−/−^ mice, but mutant cortical neurons maintain their glutamatergic identity and fail to acquire molecular features associated with GABAergic neurons. Thus, these subtype identity genes are not required for the adoption of a pan-cortical glutamatergic identity.

A different set of transcription factors, expressed in the RGCs and/or intermediate progenitors, including *Pax6*, *Tlx*, *Dmrt5*, *Dmrt3*, *Emx2*, *Ngn1*, and *Ngn2*, are essential for establishing the regional and glutamatergic identities of cortical projection neurons (Desmaris et al., 2018; Konno et al., 2019; Kroll and O’Leary, 2005; Schuurmans et al., 2004). Based on our results and previous studies, we propose that the common versus unique characteristics of cortical projection neuron subtypes are specified sequentially during development. At early stages of corticogenesis, transcription factors expressed in cortical RGCs and/or intermediate progenitors (including *Pax6*, *Tlx*, *Dmrt5*, *Dmrt3*, *Emx2*, *Ngn1*, and *Ngn2*) act in parallel pathways to ensure the generation of cortical glutamatergic projection neurons and prevent the production of ventral GABAergic neurons (Desmaris et al., 2018; Konno et al., 2019; Kroll and O’Leary, 2005; Schuurmans et al., 2004). As postmitotic cortical neurons begin to migrate and differentiate, genes such as *Fezf2*, *Tbr1*, *Sox5*, and *Satb2* repress the expression of genes associated with alternate neuronal subtype identities to establish specific subtype-specific identities. In the future, it will be necessary to rigorously test whether the proteins encoded by these genes function as transcriptional repressors or activators during development and to determine how the expression of neuronal subtype identity genes is initially activated. Answers to these questions will be invaluable for designing novel and efficient strategies for using directed differentiation or trans-differentiation methods to repair damaged brain circuits in disease and injury.

## STAR★METHODS

### RESOURCE AVAILABILITY

#### Lead contact

Further information and requests for resources and reagents should be directed to and will be fulfilled by the Lead Contact, Dr. Bin Chen (bchen@ucsc.edu).

#### Materials availability

The *Tle4*^*LacZ*^ *and Fezf2-EnR* mouse lines will be deposited to the Jackson Laboratory. All unique/stable reagents generated in this study are available from the Lead Contact, but we may require a payment and/or a completed Materials Transfer Agreement if there is potential for commercial application.

#### Data and code availability

The RNA-seq data for the *Fezf2*^−/−^, *Fezf2*^−/−^; *Fezf2-EnR*, and littermate control *Fezf2*^+/+^ cortices, and for the *Tle4*^*LacZ/LacZ*^ and littermate control *Tle4*^+/+^ cortices can be accessed using GEO: GSE160202 and GEO: GSE142269, respectively.

### EXPERIMENTAL MODEL AND SUBJECT DETAILS

#### Mice used in this study

Experiments were performed according to protocols approved by the Institutional Animal Care and Use Committee at University of California at Santa Cruz and at University of Arizona College of Medicine Phoenix, and were performed in accordance with institutional and federal guidelines. Experiments performed at Fudan University were in accordance with institutional guidelines.

We generated the *Tle4*^*LacZ*^ allele by inserting a *LacZ-ires-Plap* cassette in the intron after the exon 4 of the *Tle4* gene, using the targeted gene trap strategy (Friedel et al., 2005). Southern hybridization was performed to screen the E14a ES cell clones and identify the correct targeting.

The bacterial artificial chromosome (BAC) clone RP23-141E17 was modified by inserting the cDNA encoding the ENGRAILED transcriptional repressor domain (EnR) fused with the DNA binding domain of FEZF2, followed by the SV40 polyadenylate site (*Fezf2-EnR*), at the start codon of the mouse *Fezf2* gene. The BAC DNA was purified and sequenced and used for injection to generate the *Fezf2-EnR* transgenic mouse line.

The day of the vaginal plug detection was designated as E0.5. The day of birth was designated as P0. The genders of the embryonic and early postnatal mice were not determined.

The following mice were used in this study:

*Fezf2*^+/+^, *Fezf2*^+/−^ and *Fezf2*^−/−^ mice: P0, P7, and adult, both male and female mice were used.
*Fezf2*^*flox*/+^ and *Fezf2*^*Flox/Flox*^ mice: adult, both male and female mice were used.
*Nex-Cre* mice: adult, both male and female mice were used.
*Nex-Cre; Fezf2*^+/−^ and *Nex-Cre; Fezf2*^−/*Flox*^ mice: P0 and P7, both male and female mice were used.
*Fezf2-EnR* mice: adult, both male and female mice were used.
*Fezf2*^−/−^; *Fezf2-EnR* mice: P0, P7, and adult, both male and female mice were used.
*Tle4*^+/+^*, Tle4*^+/*LacZ*^ and *Tle4*^*LacZ/LacZ*^ mice: P0, P7, adult, both male and female mice were used.
*Tle4*^*LacZ/LacZ*^; *Fezf2-EnR* mice: P0, P7, adult, both male and female mice were used.

#### Cell lines used in this study

Neuro-2a (ATCC CCL-131) cells were used in this study for protein co-immunoprecipitation experiments. Cells were cultured at 37°C with 5% CO2 in 145 mm culture dishes (Greiner, #639960).

### METHOD DETAILS

#### PLAP staining

Human placental alkaline phosphatase (PLAP) staining was performed as described previously (Chen et al., 2005a). P7 mice were anesthetized and 4% paraformaldehyde was delivered via trans-cardiac perfusion. Brains were post-fixed in 4% paraformaldehyde for 24 hours at 4°C and then immersed in 30% sucrose in PBS for 24 hours. Brains were then frozen and sectioned into 50 μm sections using a sliding microtome (Thermo Scientific, Microm HM 430). Sections were washed 3 times in PBS, immersed in a 1:50 solution of NBT/BCIP (Roche, 11681451001) in 0.1M Tris-HCl pH 9.5, 0.1M NaCl, and then incubated at 37°C for 4 hours. Sections were then washed in PBS with 0.3% Triton X-100 5 times over the course of 2 hours at 37°C to remove background. Sections were then mounted in Fluoromount-G.

#### Immunohistochemistry

4% paraformaldehyde was delivered to mice via trans-cardiac perfusion. Brains were post-fixed in 4% paraformaldehyde, 0.1% saponin, and PBS for 24 hours at 4°C, followed by cryoprotection in 30% sucrose in PBS. Immunohistochemistry was performed using standard protocols. Briefly, twenty-five-μm-thick brain sections were permeabilized with 0.03% Triton X-100 in PBS for 30 min. Slides were then immersed in citrate buffer (10mM citric acid monohydrate, 0.05% Tween-20, pH 6.0), brought to a boil in a microwave and rested for 1 hour at RT. Slides were then incubated in a blocking buffer (5% donkey serum, 0.03% Triton X-100 in PBS) for 30 minutes. Blocking buffer was removed, and the sections were incubated with primary antibodies (diluted in the blocking buffer) for 24 hours at 4°C. The following primary antibodies were used in this study: GFP (Chicken, Aves Labs GFP-1020), BCL11B (Rat, Abcam ab18465), TBR1 (Rabbit, Abcam ab31940), SOX5 (Rabbit, Abcam ab94396), FEZF2 (Rabbit, IBL F441), TLE4 (Mouse, Santa Cruz Biotechnology sc-365406), FOXP2 (Rabbit, Abcam ab16046), ZFPM2 (Rabbit, Santa Cruz Biotechnology), B-GAL (Chicken, Abcam ab9361), activated caspase 3 (Rabbit, Cell Signaling Technology #9661), BHLHB5 (Goat, Santa Cruz Biotechnology sc-6045), SATB2 (Rabbit, Abcam ab34735), FOSL2 (Rabbit, Sigma HPA004817), and GAPDH (Covance, MMS-580S), The sections were washed in PBS, and incubated with secondary antibodies conjugated to Alexa 488, Alexa 546, or Alexa 647 for 2 hours at room temperature. Secondary antibodies were from Jackson ImmunoResearch and Invitrogen. Finally, the sections were counterstained with DAPI for 5min before being mounted in Fluoromount-G.

#### Protein co-immunoprecipitation

Neuro-2a (ATCC CCL-131) cells were cultured in 145 mm tissue culture treated dishes (Greiner, #639960) at 37°C with 5% CO2 and transfected with Lipofectamine 3000 reagent (Thermo, #L3000001) with the following plasmid combinations: Fezf2-his-myc + Tle4-flag-myc + GFP-his-myc, Fezf2-his-myc + GFP-his-myc, Tle4-flag-myc + GFP-his-myc, or GFP-his-myc alone. 24 hours later, cells were harvested, and nuclear extract was isolated using the Active Motif Nuclear Complex Co-IP kit (cat. #54001). The extracts were immunoprecipitated overnight at 4°C with either Flag-tagged beads (Sigma, #M8823) or GFP antibody (Rabbit, Invitrogen A11122) and eluted with 0.1M glycine, pH 2.5 at RT for 30 minutes with occasional agitation. The samples were denatured at 100°C for 5 minutes in 5x sample buffer, run on an 8% SDS-PAGE at 70V for 2 hours, transferred to PVDF (Sigma, IPVH85R) at 150mA for 90 minutes, and blocked for one hour in 1% non-fat dry milk-TBST. 2ug of Fezf2 (Rabbit, IBL F441), Tle4 (Mouse, Santa Cruz Biotechnology sc-365406), GFP (Chicken, Aves Labs GFP-1020), or Myc (Goat, Abcam ab9132) primary antibodies were added and incubated overnight at 4°C on an orbital shaker. The blot was developed with Li-Cor secondary antibodies (#926-32212, 926-32214, 926-68073, 926-68072) or Alexa 488 for one hour and images were processed using ImageStudioLite.

#### Western blotting

P7 cortices were dissected in ice cold 1X PBS supplemented with cOmplete Mini, EDTA-free protease inhibitor tablets (Roche, 04 693 159 001) and transferred to RIPA buffer for 20 minutes on ice. The tissue was homogenized by pushing through 25G and 27G needles sequentially, 3 times each. Cell homogenate was centrifuged at 14,000 g for 10 minutes at 4°C. The supernatant was removed, denatured at 100°C for 5 minutes in 5x sample buffer, run on an 8% SDS-PAGE at 70V for 2 hours, transferred to PVDF (Sigma, IPVH85R) at 150mA for 90 minutes, blocked for one hour in 1% non-fat dry milk-TBST, and immunoblotted overnight with 2ug of FEZF2 (Rabbit, IBL F441) primary antibody. The blot was developed with Li-Cor Donkey anti Rabbit secondary antibody (#926-68073) for one hour and images were processed using ImageStudioLite.

#### *In situ* hybridization

*In situ* hybridization was performed using a previously published protocol (Guo et al., 2013). In brief, digoxigenin-labeled probes used in this study were made from cDNAs amplified by PCR using the following primers:

**Table:** 

Gene	Forward Primer 5′ → 3′	Reverse Primer 5′ → 3′	Source
*Tcerg1l*	CTCTCCCCACTGTGGTATTAGC	CAGAACTATTTCCCCTCGTGAC	This paper
*Ldb2*	CACCTGATTACGCTGTCCATAG	AAGTTCAACACACGAGGGAGAT	This paper
*Ctgf*	AAATCGCCAAGCCTGTCAAG	GGCACTGTGCGCTAATGAAC	This paper
*Cryab*	CTCAGCCCTGCCTGTGTT	ATCTGGGCCAGCCCTTAG	This paper
*Ephb1*	CACATCCATCTCCCTTTGCT	TCCAGAAACCCTTTCCCTCT	(Lodato et al., 2014)
*Kif26a*	TCCTCAGCTCCAGACTCCAT	GCGACAGTCTTTCCATCTCC	(Lodato et al., 2014)
*Wnt7b*	ACGCAATGGTGGTCTGGT	AAGGGCCTGAGGAAATGG	Allen Brain Atlas

Amplified DNA fragments were ligated into pGEM-T Easy (Promega) plasmids, transformed into competent *E. coli* cells, and plated overnight on LB+Agar+Ampicillin plates. Colonies were picked, grown overnight in 3 mL LB+Ampicillin, and purified via miniprep kits (Sigma-Aldrich). Purified plasmids were sequenced to ensure sequence fidelity, and to determine insert orientation. Plasmids were then linearized with restriction enzymes from New England Biotech, and *in vitro* transcription reactions were performed with either T7 (NEB) or Sp6 (Promega) RNA polymerases, depending on insert orientation, in the presence of DIG-labeled nucleotides (Sigma-Aldrich). Tissue was prepared as previously described (Guo et al., 2013), and treated with DIG-labeled probes overnight at 65°C. Slides were developed with NBT/BCIP stock solution (Sigma-Aldrich).

#### EdU labeling

Timed pregnant *Tle4*^+/*LacZ*^ mice were injected with a single dose of EdU (50mg/kg body weight; Thermo Fisher Scientific, E10187) at E12.5 or E13.5. Brains were collected at P7. EdU was detected via a click-chemistry reaction containing the following reagents per 1 mL of reaction: 950ul 100mM Tris PH 7.4, 40ul 100 mM CuSO4, 10ul 200 mg/mL sodium ascorbate, and 1ul azide 488 or 555. *Tle4*^*LacZ/LacZ*^ and littermate *Tle4*^+/+^ control mice were analyzed.

#### Anterograde tracing using AAV

0.25 μl AAV2-CMV-mCherry virus (Vector Biosystems Inc.) were injected into the M1, S1 or V1 of *Tle4*^*LacZ/LacZ*^ and littermate control *Tle4*^+/*LacZ*^ or *Tle4*^+/+^ mice at P21. The brains were collected at P35 and sectioned at 50-μm thickness.

#### Retrograde tracing

Retrograde tracing was performed using Alexa Fluor 555-conjugated cholera toxin subunit β (CTB) injections. 8 mg/ml CTB in PBS was used for all injections, and CTB solution was injected through a pulled glass pipet attached to a Picospritzer III (Parker). P4 mice were anesthetized, the pyramidal decussation was identified visually, and 1 μl CTB was injected. 0.2 to 0.5 μl CTB was injected into S1 in anesthetized P4 mice and injection sites were confirmed after brain collection at P7. Corticothalamic neurons were labeled by CTB injection (0.2 μl) into the thalamus at P21 (coordinates: A/P −1.3 mm, M/L 3 mm, Z 3.15 mm) and injection sites were confirmed after brain collection at P28.

#### Cloning of the pCAG-Fezf2, pCAG-Fezf2-EnR, and the pCAG-Fezf2-VP16 expression plasmids

The cloning of *pCAG-Fezf2* plasmid was reported previously (Chen et al., 2005a). The EnR and VP16 plasmids were obtained from Dr. Thomas Jessell (Columbia University). The cDNA for DNA binding domains of FEZF2 was amplified using primers 5′-GATCGAATTCTCAGCTCTGAACTGTCCTGGCTAGGTC-3′ and 5′-GATCGGATCCGCCGCCGCCATGGAGCCCCGGCCTGCTGCGTTAG AGGC-3′. The cDNA for the EnR domain was amplified using primers 5′-GATCGATATCAAGCTTGGGCTGCATAGATCCCAG-3′ and 5′-GATCGGATCCGCCGCCACCATGGAGTTCCGCGATGCCCTGGAGGATCGC-3′. The cDNA for VP16 domain was amplified using primers 5′-GATCGGATCCGCCGCCACCATGGCCCCCCCGACCGATGTCAGCCT-3′ and 5′-GATCGATATCCCCACCGTACTCGTCAATTCCAA-3′. The amplified DNA fragments were ligated into pCAG vector, using NotI and XhoI restriction sites. Sanger DNA sequencing was performed to ensure no mutation was generated during the cloning.

#### In utero electroporation

In utero electroporation experiment was performed according to a published protocol (Chen et al., 2005a). In utero electroporation (IUE) of wild-type CD-1 embryos was performed at E15.5. Plasmids *pCAG-Fezf2*, *pCAG-Fezf2-EnR*, or *pCAG-Fezf2-VP16* were mixed with *pCAG-EGFP* (Addgene #11150) (final concentration of 1–2 μg/μl at a molecular ratio of 3:1, 0.5 μL each embryo) and 0.05% Fast Green (Sigma), and injected into the lateral ventricle of embryos using a beveled pulled glass micropipette. The control brains were electroporated with *pCAG-EGFP* plasmids alone. Five electrical pulses (duration: 50 ms) were applied at 35V across the uterine wall with a 950 ms interval between pulses. Electroporation was performed using a pair of 7-mm platinum electrodes (BTX, Tweezertrode 45-0488, Harvard Apparatus) connected to an electroporator (BTX, ECM830). The electroporated brains were collected at P5.

#### Image acquisition and analysis

Images for quantitative analyses were acquired with a Zeiss 880 confocal microscope. Laser power and gain were adjusted until < 1% of pixels were saturated. Cell counting was performed on single z-slices with FIJI. Z-slices were divided into 500 μm or 250 um wide regions and split into equally sized bins. Individual channels were adjusted with auto threshold “Moments,” or a manual threshold was applied to discern BCL11B high versus low expressing cells. The dilate, erode, and watershed functions were sequentially used before particles were analyzed with a circularity of 0.3–1.0 and size exclusion of > 1 μm. Brightfield images were acquired with a Zeiss AxioImager Z2 widefield microscope with a Zeiss AxioCam 506 (color) camera.

Statistical analysis was performed using GraphPad Prism 5.0, or R. Only single Z-slice confocal images were used in cell quantifications. For each brain, the number of marker^+^ cells in the cortex were quantified in a 500- or 250-mm-wide region from 3 sections each for S1, M1 and V1 areas. Care was taken to match the anterior-posterior, medial-lateral positions for the chosen areas between the mutant and control genotypes. For each genotype and each age, 3 different brains were analyzed. Data are shown as mean ± SEM and statistical significance for multiple comparisons was determined using the ordinary one-way ANOVA test followed by Tukey’s multiple comparisons test. Statistical significance for single comparisons was determined using the unpaired t test. Significance was set as * for p < 0.05, ** for p < 0.01, *** p < 0.001 for and ****p < 0.0001 all significance tests.

#### RNA-seq analysis of Tle4^LacZ/LacZ^, Fezf2^−/−^, and Fezf2^−/−^; Fezf2-EnR cortices at P0

Cortices were dissected from P0 *Tle4*^*LacZ/LacZ*^ (n = 3 mice) and littermate control *Tle4*^+/+^ (n = 3) mice, P0 *Fezf2*^−/−^ (n = 4), *Fezf2*^−/−^; *Fezf2-EnR* (n = 2), and littermate *Fezf2*^+/+^ mice (n = 4). Total RNA from each pair of cortical hemispheres was isolated using the RNAe-say kit (QIAGEN) and used to prepare RNA-seq libraries (Illumina RNA Truseq Library Prep protocol). Libraries were paired-end (50 nucleotides per end) sequenced on the Illumina Hiseq2000 platform. The sequences were processed and analyzed for differential expression as previously described (Betancourt et al., 2014). The RNA-seq data for the *Fezf2*^−/−^, *Fezf2*^−/−^; *Fezf2-EnR*, and control *Fezf2*^+/+^ cortices, and for the *Tle4*^*LacZ/LacZ*^ and control *Tle4*^+/+^ cortices can be accessed using GSE160202 and GSE142269, respectively.

#### Electrophysiology and neuronal morphology

Whole cell recording was conducted in the primary somatosensory cortex (V1). To label layer 6 corticothalamic neurons, 50nl of retrobeads (Lumafluor) were injected into the POM nucleus unilaterally at least 24h prior to recording. 350-μm slices were made after a block cut of the posterior brain with a 45° angle to the mid-sagittal plane. Slices were cut in ice-cold ACSF (containing 126 mM NaCl, 2.5 mM KCl, 26 mM NaHCO_3_, 2 mM CaCl_2_, 1 mM MgCl_2_, 1.25 mM NaH_2_PO_4_, and 10 mM glucose saturated with 95% O_2_ and 5% CO_2_). Slices were incubated at 32°C for 30 min before being transferred to the recording chamber. Beads^+^ neurons with soma in layer 6 were identified under a 60X objective (NA = 0.9). Only neurons with their soma at least 50 μm below the slice surface were targeted for whole cell recordings. The internal electrode solution contains: 130 mM K-gluconate, 10 mM HEPES, 4 mM KCl, 0.3 mM GTP-Na, 4 mM ATP-Mg, 2 mM NaCl,1 mM EGTA and 14 mM phosphocreatine (pH 7.2, 295–300 mOsm). 0.15% (W/V) biocytin was added when neuron morphology data were desired.

Neuronal signals were amplified using a MultiClamp 700B amplifier (Molecular Devices, Forster City, CA), low-pass filtered at 1 kHz (current) or 10 kHz (voltage signals), and digitized at 20 kHz using a Digidata 1440A interface and pClamp 10.6 (Molecular Devices). mEPSCs were measured with D-AP5 (50 μM, Tocris) and tetrodotoxin (TTX, 1 μM, Tocris) included in the ACSF. To measure mIPSCs, TTX (1 μM) and CNQX (10 μM) were included and a symmetrical [Cl^−^] electrode internal solution (containing: 125 mM KCl, 2.8 mM NaCl, 2 mM MgCl_2_, 2 mM Mg^2+^-ATP, 0.3 mM Na_3_GTP, 10 mM HEPES, 1 mM EGTA and 10 mM phosphocreatine, pH 7.25, ~300 mOsm) was used. In experiments where neuronal excitability was measured, a series of current steps (−100 to 500pA in 50 pA increment) were injected, and numbers of AP firing were manually quantified.

To reconstruct neuronal morphologies, slices were fixed in 4% PFA overnight, followed by incubation with avidin-Alexa 488 (Invitrogen) for 24 h in PBS containing 0.2% Triton X-100. Slices were washed and mounted on slides with a 350-μm spacer to prevent crushing the tissue. Neuronal dendritic arbors were acquired by collecting Z stack images on a confocal microscope (Zeiss LSM 710). Maximal projection images were imported into FIJI/ImageJ, and neurite arborization and Sholl analysis (Sholl, 1953) were done using the Simple Neurite Tracer plugin. Due to the length of apical dendrites, dendrites were frequently cut off. Therefore, only basal dendrites were used for Sholl analysis. Morphometric features extracted included dendritic arbor, length, and number of intersections at various distances from soma. For dendritic spine analyses, Z stacks of spines from the basal dendrites (100–450 μm away from soma) were collected with a 63x objectives (Plan-Apochromat, NA 1.4). 512 × 3 512 pixels with 4 × digital zoom and 0.2 μm Z step size were used for Z stack acquisition. Imaris software (V8.02, Bitplane, South Windsor, CT) was used to measure spine head diameter, length, and density (Peng et al., 2016).

### QUANTIFICATION AND STATISTICAL ANALYSIS

The statistical details of the experiments can be found in the figure legends and Method details section under the experiments. All n values and what n represents are listed in the figure legends, and all p values obtained are listed in the figure legends. GraphPad Prism version 8 was used to perform statistical tests in this study. The statistical tests used for each experiment are indicated in the figure legends.

## Supplementary Material

1

## Figures and Tables

**Table:** KEY RESOURCES TABLE

REAGENT or RESOURCE	SOURCE	IDENTIFIER
Antibodies		
Chicken anti-GFP polyclonal	Aves Labs	GFP-1020; RRID:AB_2307313
Rat anti-BCL11B monoclonal	Abcam	ab18465; RRID:AB_2064130
Rabbit anti-TBR1 polyclonal	Abcam	ab31940; RRID:AB_2200219
Rabbit anti-SOX5 polyclonal	Abcam	ab94396; RRID:AB_10859923
Rabbit anti-FEZF2 polyclonal	IBL	F441; RRID:AB_529237
Mouse anti-TLE4 monoclonal	Santa Cruz Biotechnology	sc-365406; RRID:AB_10841582
Rabbit anti-FOXP2 polyclonal	Abcam	ab16046; RRID:AB_2107107
Rabbit anti-ZFPM2 polyclonal	Santa Cruz Biotechnology	sc-10755; RRID:AB_2218978
Chicken anti-B-GAL polyclonal	Abcam	ab9361; RRID:AB_307210
Rabbit anti-Activated Caspase 3 polyclonal	Cell Signaling Technology	9661; RRID:AB_2341188
Goat anti-BHLHB5 polyclonal	Santa Cruz Biotechnology	sc-6045; RRID:AB_2065343
Rabbit anti-SATB2 polyclonal	Abcam	ab34735; RRID:AB_2301417
Rabbit anti-Fosl2 polyclonal	Sigma	HPA004817; RRID:AB_1849014
Mouse anti-GAPDH monoclonal	Biolegend	919501; RRID:AB_2565211
Donkey anti-Chicken Alexa Fluor 488	Jackson ImmunoResearch Labs	703-545-155; RRID:AB_2340375
Donkey anti-Mouse Alexa Fluor 488	Invitrogen	R37114; RRID:AB_2556542
Donkey anti-Rabbit Alexa Fluor 488	Invitrogen	A-21206; RRID:AB_2535792
Donkey anti-Goat Alexa Fluor 488	Invitrogen	A-11055; RRID:AB_2534102
Donkey anti-Rat Alexa Fluor 488	Invitrogen	A-21208; RRID:AB_141709
Goat anti-Chicken Alexa Fluor 546	Invitrogen	A-11040
Donkey anti-Mouse Alexa Fluor 594	Jackson ImmunoResearch Labs	715-585-150; RRID:AB_2340854
Donkey anti-Rabbit Alexa Fluor 546	Invitrogen	A-10040; RRID:AB_2534016
Donkey anti-Goat Alexa Fluor 546	Invitrogen	A-11056
Donkey anti-Rat Alexa Fluor 555	Abcam	Ab150154; RRID:AB_2813834
Donkey anti-Chicken Alexa Fluor 647	Jackson ImmunoResearch Labs	703-606-155; RRID:AB_2340380
Donkey anti-Mouse Alexa Fluor 647	Invitrogen	A-31571; RRID:AB_162542
Donkey anti-Rabbit Alexa Fluor 647	Invitrogen	A-31573; RRID:AB_2536183
Donkey anti-Goat Alexa Fluor 647	Invitrogen	A-21447; RRID:AB_141844
Donkey anti-Rat Alexa Fluor 647	Jackson ImmunoResearch Labs	712-605-153; RRID:AB_2340694
Donkey anti-Mouse HRP	Invitrogen	A16011; RRID:AB_2534685
Donkey anti-Rabbit HRP	Invitrogen	A16035; RRID:AB_2534709
Rabbit anti GFP	Invitrogen	A1122; RRID:AB_221569
Goat anti MYC	Abcam	Ab9132; RRID:AB_307033
Donkey anti-Mouse IgG IRDye 800	Li-Cor	926-32212; RRID:AB_621847
Donkey anti-Goat IgG IRDye 800	Li-Cor	926-32214; RRID:AB_621846
Donkey anti-Rabbit IgG IRDye 680	Li-Cor	926-68073; RRID:AB_10954442
Donkey anti-Mouse IgG IRDye 680	Li-Cor	926-68072; RRID:AB_10953628
Anti FLAG-M2 Magnetic Beads	Sigma	M8823; RRID:AB_2637089
Bacterial and virus strains		
AAV2-CMV-mCherry virus	Vector Biosystems Inc.	#7104
Biological samples		
Mouse cortex	This paper	N/A
Chemicals, peptides, and recombinant proteins		
EdU	Thermo Fisher	E10187
(+)-Sodium L-ascorbate	Sigma-Aldrich	A7631
Copper (II) Sulfate (CuSO4)	Sigma-Aldrich	451657
Rhodamine Azide	Invitrogen	A20012
Alexa Fluor 488 Azide	Invitrogen	A10266
Cholera Toxin Subunit B, Alexa Fluor 555 conjugate	Invitrogen	C22843
Saponin	Sigma-Aldrich	470366
Paraformaldehyde (PFA)	MP Biomedicals	150146
Citric Acid Monohydrate	Sigma-Aldrich	C0706
Horse Serum	GIBCO	16050-114
T7 Polymerase	New England Biotechnology	M0251
SP6 Polymerase	Promega	P1085
DIG-Labeled Nucleotides	Sigma-Aldrich	11277073910
NBT/BCIP stock solution	Sigma-Aldrich	11681451001
SpeI Restriction Enzyme	New England Biotechnology	R3133
SalI Restriction Enzyme	New England Biotechnology	R0138
NcoI Restriction Enzyme	New England Biotechnology	R0193
NotI Restriction Enzyme	New England Biotechnology	R0189
XhoI Restriction Enzyme	New England Biotechnology	R0146
Trypan Blue Stain (0.4%)	GIBCO	15250-061
Red Retrobeads	Lumafluor	R170
Avidin Alexa Fluor 488	Invitrogen	A21370
Critical commercial assays		
GenElute HP Plasmid Miniprep Kit	Sigma-Aldrich	NA0160
Qiaquick PCR Purification Kit	QIAGEN	28106
RNeasy Plus Mini Kit	QIAGEN	74134
TruSeq RNA Library Prep Kit	Illumina	RS-122-2001
Nuclear Complex Co-IP kit	Active Motif	54001
Deposited data		
*Fezf2*^−/−^, *Fezf2*^−/−^; *Fezf2-EnR* and control RNA-seq data	This paper	GEO: GSE160202
*Tle4*^*LacZ/LacZ*^ and control RNA-seq data	This paper	GEO: GSE142269
Experimental models: cell lines		
Neuro-2a	ATCC	CCL-131
Experimental models: organisms/strains		
Mouse: *Fezf2*^−^	Chen et al., 2005a	University of California, Santa Cruz
Mouse: *Fezf2^Flox^*	Han et al., 2011	Yale University
Mouse: *Fezf2-EnR*	This Paper	N/A
Mouse: *Tle4*^*LacZ*^	This Paper	N/A
Mouse: *Nex-Cre*	Goebbels et al., 2006	Max-Planck-Institute of Experimental Medicine
Oligonucleotides		
Forward primers for genotyping *Tle4* wildtype allele: GAGATGTGGCTAC AGAAGAGGTTCAGAGAC	This Paper	N/A
Reverse primers for genotyping *Tle4* wildtype allele: ATCTGCCCC TTGCTATTCCTGCTTGCTCTC	This Paper	N/A
Forward primers for genotyping *Tle4*^*LacZ*^ allele: TGCTCTCCCACAAGCTCGCTTGTCGTTCAG	This Paper	N/A
Reverse primers for genotyping *Tle4*^*LacZ*^ allele: AAGAGGGCTCTGTCCTCCAGTCTCCTCCAC	This Paper	N/A
Forward primers for genotyping *Fezf2* wildtype allele: TTGAATGCAAATGGGTGACCGGGCCG	This Paper	N/A
Reverse primers for genotyping *Fezf2* wildtype allele: GTTTTAGAAGTGGCCGGTGACGCTCC	This Paper	N/A
Forward primers for genotyping *Fezf2*^−^ allele: CACCCCGGTGAACAGCTCC TCGCCCTTGCTCACCAT	This Paper	N/A
Reverse primers for genotyping *Fezf2*^−^ allele: CTGCATGGCTCGGAACG CATCTCCTTGGCGGTGGGGGAAAGAG	This Paper	N/A
Forward primers for genotyping *Fezf2-EnR* allele: CAAAATCGGTTACGGTTGAGTAATA	This Paper	N/A
Reverse primers for genotyping *Fezf2-EnR* allele: ACCATGCCACTTCCCTTCTCAG	This Paper	N/A
Forward primers for genotyping *Fezf2*^*Flox*^ allele: TGCCTTGTACACCTTTCTCT	Han et al., 2011	Yale University
Reverse primers for genotyping *Fezf2*^*Flox*^ allele: GAGACCTAGGCAAGGGACAGT	Han et al., 2011	Yale University
Forward primer for genotyping *Nex-Cre* allele: GAGTCCTGGAATCAGTCTTTTTC	This Paper	N/A
Reverse primer for genotyping *Nex-Cre* allele: CCGCATAACCAGTGAAACAG	This Paper	N/A
Forward primers for Tcerg1l *in situ* hybridization probe template: CTCTCCCCACTGTGGTATTAGC	This Paper	N/A
Reverse primers for Tcerg1l *in situ* hybridization probe template: CAGAACTATTTCCCCTCGTGAC	This Paper	N/A
Forward primers for Ldb2 *in situ* hybridization probe template: CACCTGATTACGCTGTCCATAG	This Paper	N/A
Reverse primers for Ldb2 *in situ* hybridization probe template: AAGTTCAACACACGAGGGAGAT	This Paper	N/A
Forward primers for Ctgf *in situ* hybridization probe template: AAATCGCCAAGCCTGTCAAG	This Paper	N/A
Reverse primers for Ctgf *in situ* hybridization probe template: GGCACTGTGCGCTAATGAAC	This Paper	N/A
Forward primers for Cryab *in situ* hybridization probe template: CTCAGCCCTGCCTGTGTT	This Paper	N/A
Reverse primers for Cryab *in situ* hybridization probe template: ATCTGGGCCAGCCCTTAG	This Paper	N/A
Forward primers for Ephb1 *in situ* hybridization probe template: CACATCCATCTCCCTTTGCT	Lodato et al., 2014	N/A
Reverse primers for Ephb1 *in situ* hybridization probe template: TCCAGAAACCCTTTCCCTCT	Lodato et al., 2014	N/A
Forward primers for Kif26a *in situ* hybridization probe template: TCCTCAGCTCCAGACT CCAT	Lodato et al., 2014	N/A
Reverse primers for Kif26a *in situ* hybridization probe template: GCGACAGTCTTTCCATCTCC	Lodato et al., 2014	N/A
Forward primers for Wnt7b *in situ* hybridization probe template: ACGCAATGGTGGTCTGGT	Allen Brain Atlas	https://developingmouse.brain-map.org/experiment/show/100054743
Reverse primers for Wnt7b *in situ* hybridization probe template: AAGGGCCTGAGGAAATGG	Allen Brain Atlas	https://developingmouse.brain-map.org/experiment/show/100054743
Forward primers for VP16: gatcggatccgccgccaccatgg cccccccgaccgatgtcagcct	This Paper	N/A
Reverse primers for VP16: gatcgatatccccaccgtactcgtcaattccaa	This Paper	N/A
Recombinant DNA		
pGEM-T Easy Vector System	Promega	A1360
*pCAG-Fezf2*	Chen et al., 2008	University of California, Santa Cruz
*pCAG-Fezf2-EnR*	This Paper	N/A
*pCAG-Fezf2-VP16*	This Paper	N/A
*pCAG-EGFP*	Matsuda and Cepko, 2004	Addgene #11150
*pCMV-Tle4-Myc-DDK tag*	This paper	Origene #MR231124
Software and algorithms		
FIJI	Schindelin et al., 2012	https://imagej.net/Fiji
Adobe Illustrator	Adobe	https://www.adobe.com/products/illustrator.html
Adobe Photoshop	Adobe	https://www.adobe.com/products/photoshop.html
Imaris	Bitplane	https://imaris.oxinst.com/
Zen Imaging	Zeiss	https://www.zeiss.com/microscopy/us/products/microscope-software/zen.html
GraphPad prism v8	GraphPad	https://www.graphpad.com/scientific-software/prism/
RepeatMasker Library	Smit et al., 1996	http://repeatmasker.org/libraries/
TopHat	Trapnell et al., 2009	https://www.encodeproject.org/software/tophat/
Bowtie	Langmead et al., 2009	http://bowtie-bio.sourceforge.net/index.shtml
SamTools	Li et al., 2009	http://samtools.sourceforge.net/
DESeq	Anders and Huber, 2010	https://bioconductor.org/packages/devel/bioc/vignettes/Glimma/inst/doc/DESeq2.html
Database for Annotation, Visualization and Integrated Discovery (DAVID)	Huang et al., 2007	https://david.ncifcrf.gov/
pClamp 10.6	Molecular Devices	http://go.moleculardevices.com/l/83942/2015-09-08/77t9w
ImageStudioLite	Li-Cor	https://www.licor.com/bio/image-studio-lite/download
Other		
7-mm Platinum Electrodes	BTX/Harvard Apparatus	45-0488
ECM 399 Electroporation System	BTX	45-0000
Zeiss 880 Confocal Microscope	Zeiss	LSM 880
Zeiss Axio Imager Z2 Widefield Microscope	Zeiss	Axio Imager 2
Zeiss LSM 710	Zeiss	LSM 710
MultiClamp 700B amplifier	Molecular Devices	1-CV-7B
Digidata 1440A	Molecular Devices	N/A

## Highlights

- FEZF2 specifies subtype-specific fates in newly born neurons
- FEZF2 functions as a transcriptional repressor to specify cell fate
- FEZF2 specifies cell fates by repressing alternate cell-type-specific genes
- FEZF2 and TLE4 are co-repressors in corticothalamic projection neurons
