# Supplementary material for: Transcriptional repression by FEZF2 restricts alternative identities of cortical projection neurons
Source: Cell Rep. Author manuscript; Available in PMC 2021 Aug 2. (PMC8327856; doi:10.1016/j.celrep.2021.109269)
Supplement: 1 [file NIHMS1717853-supplement-1.pdf]

Cell Reports, Volume *35*

Supplemental information

Transcriptional repression by FEZF2 restricts alternative identities of cortical projection neurons

Jeremiah Tsyporin, David Tastad, Xiaokuang Ma, Antoine Nehme, Thomas Finn, Liora Huebner, Guoping Liu, Daisy Gallardo, Amr Makhamreh, Jacqueline M. Roberts, Solomon Katzman, Nenad Sestan, Susan K. McConnell, Zhengang Yang, Shenfeng Qiu, and Bin Chen

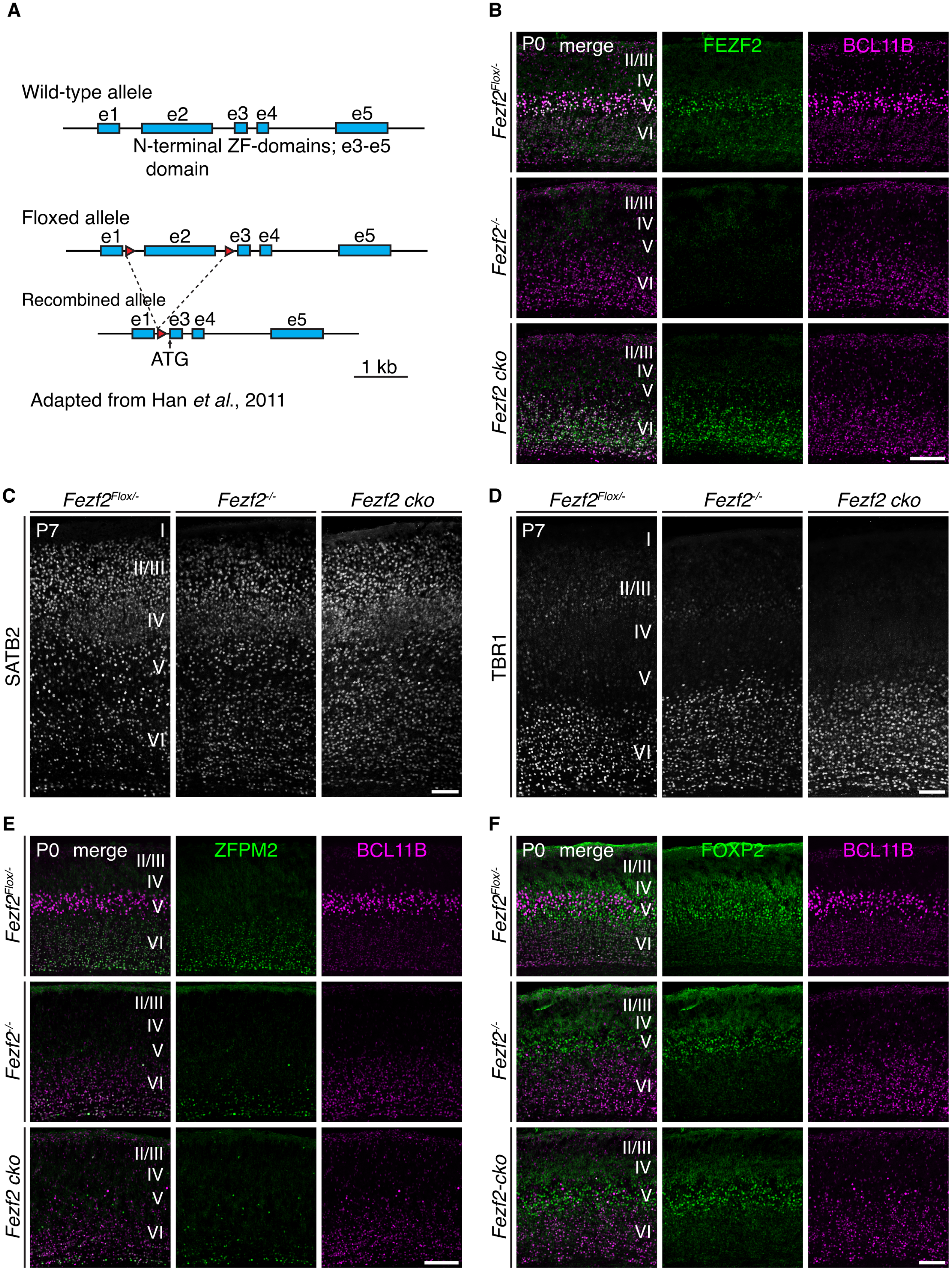
A
Wild-type allele
e1
e2
e3
e4
e5
N-terminal domain
ZF-domains; e3-e5
Floxed allele
e1
e2
e3
e4
e5
Recombined allele
e1
e3
e4
e5
ATG
1 kb
Adapted from Han et al., 2011
B
P0
merge
FEZF2
BCL11B
Fezf2Flox/-
Fezf2-/-
Fezf2 cko
II/III
IV
V
VI
C
Fezf2Flox/-
Fezf2-/-
Fezf2 cko
SATB2
P7
I
II/III
IV
V
VI
D
Fezf2Flox/-
Fezf2-/-
Fezf2 cko
TBR1
P7
I
II/III
IV
V
VI
E
P0
merge
ZFPM2
BCL11B
Fezf2Flox/-
Fezf2-/-
Fezf2 cko
II/III
IV
V
VI
F
P0
merge
FOXP2
BCL11B
Fezf2Flox/-
Fezf2-/-
Fezf2-cko
II/III
IV
V
VI

Figure S1. The *Fezf2*$^{-/-}$ and *Fezf2 cko* mice show similar molecular defects in the cortical projection neurons.

Related to Figure 1.

A. The recombined *Fezf2*^{Flox} allele encodes a truncated FEZF2 protein consisting of the zinc finger DNA binding domain alone. **B**. Immunostaining using a C-terminal FEZF2 antibody shows the complete absence of FEZF2 protein in the *Fezf2*$^{-/-}$ mice, and increased expression of the truncated FEZF2 protein in layer 6 in the *Fezf2 cko* mice. **C**. SATB2 protein expression is increased in the deep layers of *Fezf2*$^{-/-}$ and *Fezf2 cko* cortices. **D**. TBR1 protein expression is increased in the layer 5 of *Fezf2*$^{-/-}$ and *Fezf2 cko* cortices. **E-F**. ZFPM2 (**E**) and FOXP2 (**F**) expression is reduced in layer 6 of *Fezf2*$^{-/-}$ and *Fezf2 cko* cortices. Scale bars: 100 µm.

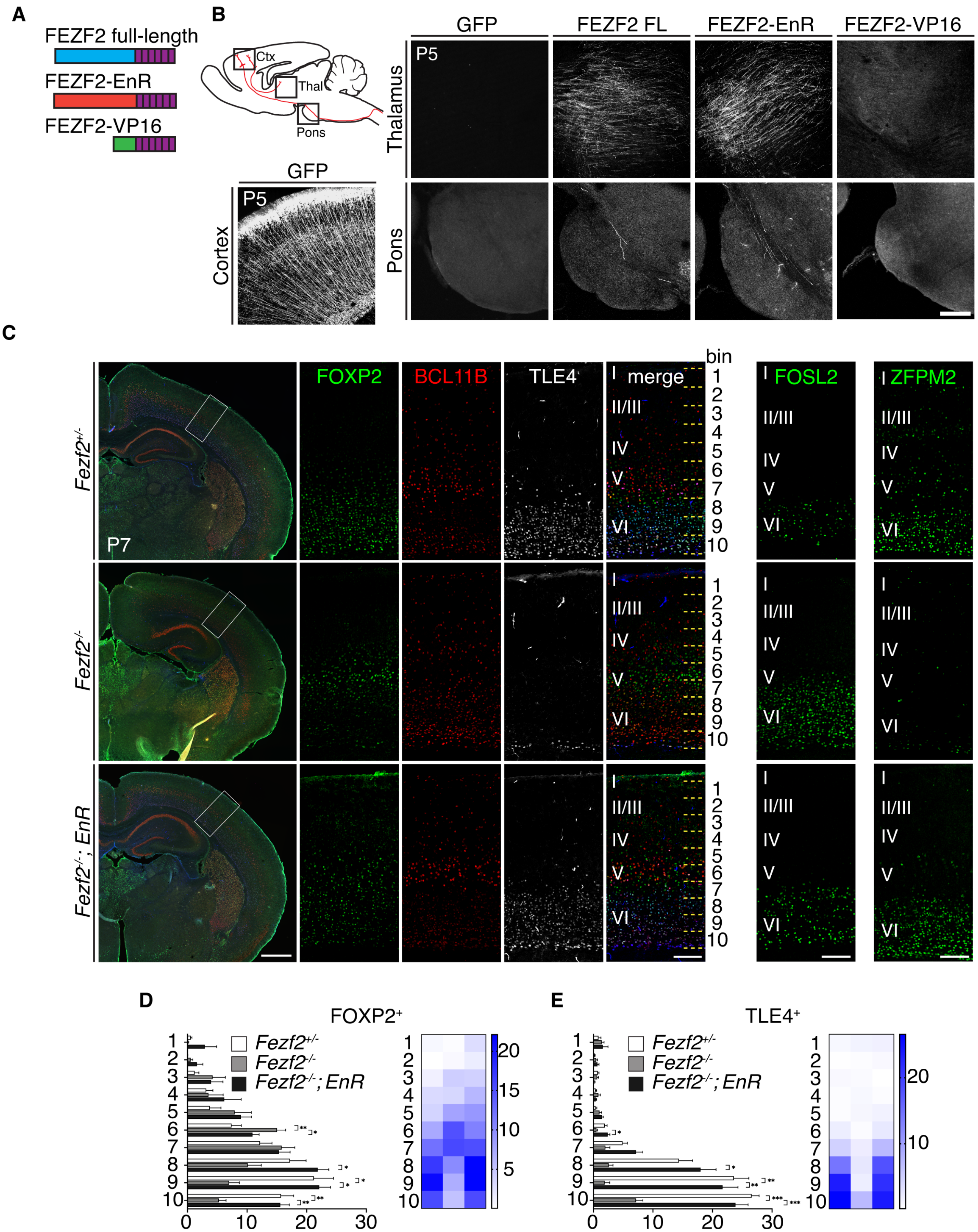

A
FEZF2 full-length
FEZF2-EnR
FEZF2-VP16
B
Ctx
Thal
Pons
GFP
P5
Cortex
GFP
FEZF2 FL
FEZF2-EnR
FEZF2-VP16
Thalamus
P5
Pons
C
Fezf2+/-
Fezf2-/-
Fezf2-/-; EnR
P7
FOXP2
BCL11B
TLE4
merge
bin
I
II/III
IV
V
VI
1
2
3
4
5
6
7
8
9
10
FOSL2
ZFPM2
D
FOXP2+
Fezf2+/-
Fezf2-/-
Fezf2-/-; EnR
0
10
20
30
5
10
15
20
E
TLE4+
Fezf2+/-
Fezf2-/-
Fezf2-/-; EnR
0
10
20
30
10
20

Figure S2. FEZF2 functions as a transcriptional repressor during cortical development.

Related to Figure 2.

A. Schematic representations of full-length FEZF2 protein, the FEZF2-EnR chimeric protein, and the FEZF2-VP16 chimeric protein. blue: the N-terminal half of FEZF2; purple: the 6 C2H2-type zinc finger motifs of FEZF2; red: the EnR transcriptional repressor domain; green: the VP16 transcriptional activator domain. **B**. Over-expression of full-length FEZF2, FEZF2-EnR, and FEZF2-VP16 into layer 2/3 neurons by *in utero* electroporation at E15.5, and the effect on axonal projection was assessed at P5 using GFP immunostaining. GFP labeled cells and axons in three boxed areas (cortex, thalamus and pontine nuclei) were shown. Scale bar, 200 μm. **C**. Immunostaining of FOXP2, BCL11B, TLE4 and FOSL2 in brains of P7 *Fezf2*$^{+/-}$, *Fezf2*$^{-/-}$, and *Fezf2*$^{-/-}$; *Fezf2-EnR* mice. Scale bars: low mag, 500 μm, high mag 100 μm. **D-E**. Quantifications of marker^{+} cells per 10,000 μm^2 per bin. n=3 brains per genotype, 3 sections per brain. In all graphs, error bars represent ± SEM. Statistical significance was determined using one-way ANOVA followed by post hoc Tukey's t-test (*p<0.05, **p<0.01, ***p<0.001). Heatmaps show mean numbers of cells per 10,000 um^2 for each bin.

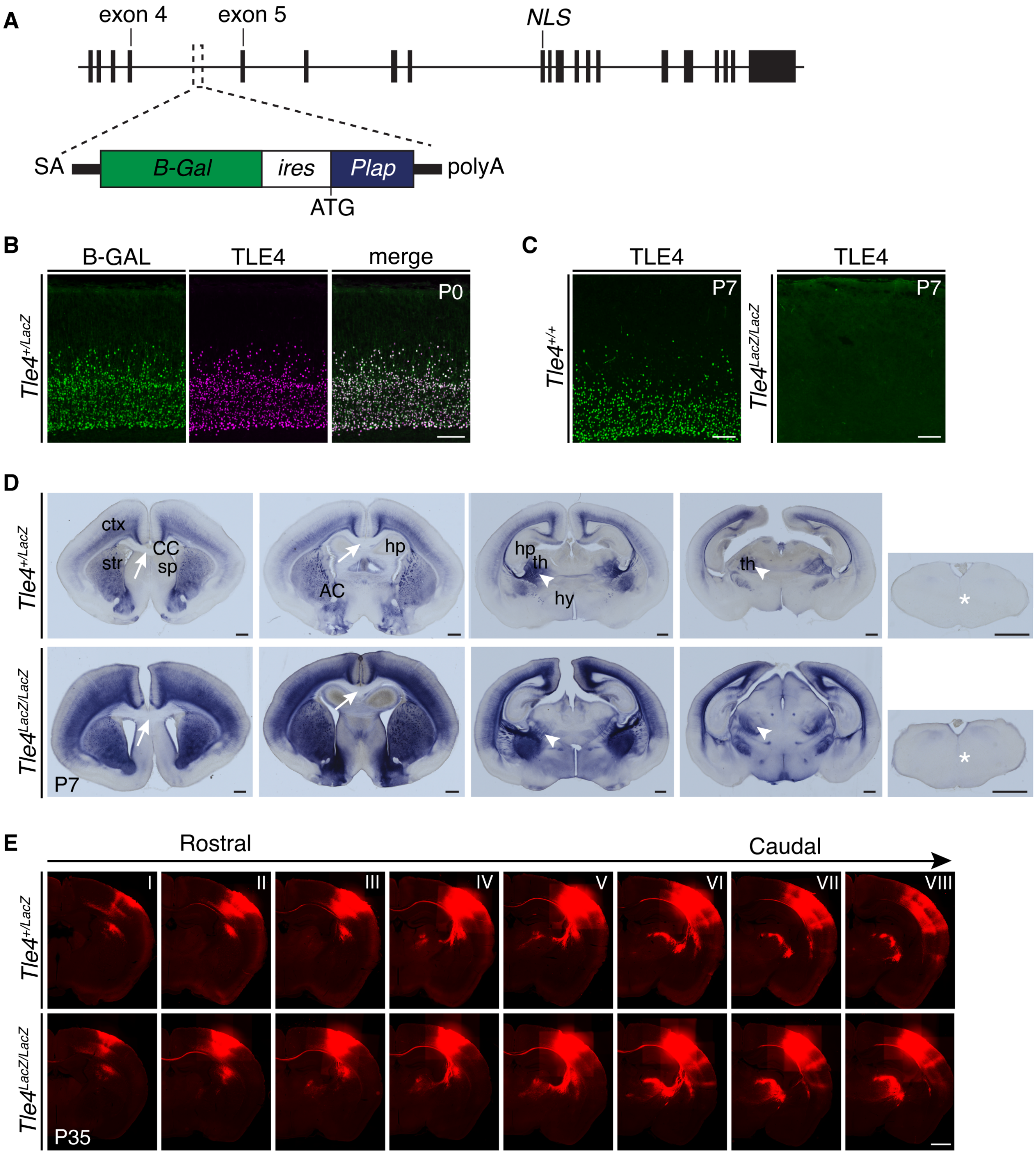
A
exon 4
exon 5
NLS
SA
B-Gal
ires
Plap
polyA
ATG
B
B-GAL
TLE4
merge
P0
Tle4+/LacZ
C
TLE4
P7
Tle4+/+
Tle4LacZ/LacZ
D
ctx
CC
str
sp
hp
AC
th
hy
P7
E
Rostral
Caudal
I
II
III
IV
V
VI
VII
VIII
P35

Figure S3. Corticothalamic neurons project axons properly into the thalamus of *Tle4*$^{LacZ/LacZ}$ mice.

Related to Figure 5.

A. Knockout strategy for *Tle4*. The SA-B-Gal-ires-Plap cassette was inserted into the intron after exon 4. *SA*: splicing acceptor; *B-Gal*: β-galactosidase; *ires*: internal ribosome entry site; *Plap*: human placental alkaline phosphatase; *NLS: nuclear localization signal.* **B**. Immunostaining showed that B-GAL recapitulates TLE4 expression pattern in the *Tle4*$^{+/LacZ}$ mice. Scale bar: 100 µm. **C**. TLE4 protein is not detected by immunostaining in the *Tle4*$^{LacZ/LacZ}$ mice. Scale bar: 100 µm. **D**. PLAP staining of P7 *Tle4*$^{+/LacZ}$ and *Tle4*$^{LacZ/LacZ}$ mice. arrows: corpus callosum; arrowheads: thalamus; *: pyramidal decussation. Scale bars for all images in **D**: 500 µm. AC: anterior commissure; ctx: cerebral cortex, CC: corpus callosum, str: striatum, sp: septum, hp: hippocampus; hy: hypothalamus; th: thalamus. **E**. AAV-mCherry was injected into the primary somatosensory cortex in the *Tle4*$^{+/LacZ}$ and *Tle4*$^{LacZ/LacZ}$ mice at P21, and brains were collected at P35. Eight consecutive 50-µm sections (I-VIII) are shown. Scale bar: 1mm.

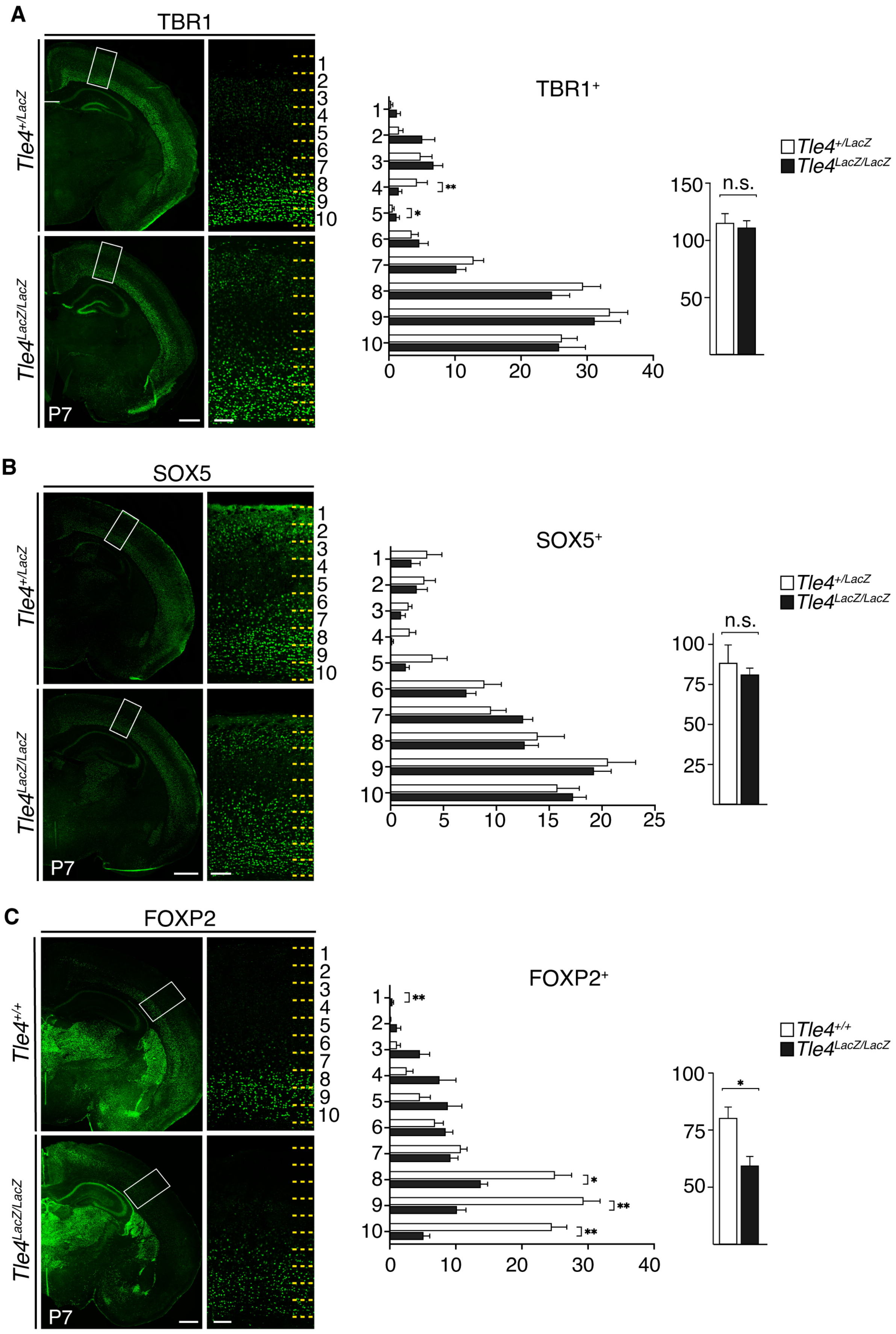
A
TBR1
Tle4+/LacZ
Tle4LacZ/LacZ
P7
TBR1+
n.s.
B
SOX5
SOX5+
C
FOXP2
Tle4+/+
FOXP2+

Figure S4. The numbers of TBR1^{+} and SOX5^{+} layer 6 neurons were not affected in *Tle4*$^{LacZ/LacZ}$ cortices, while the number of FOXP2^{+} cells was reduced.

Related to Figure 5.

A. TBR1 staining, quantification by bin and the total numbers of TBR1^{+} cells in *Tle4*$^{+/LacZ}$ and *Tle4*$^{LacZ/LacZ}$ cortices. **B**. SOX5 staining, quantification by bin and the total numbers of SOX5^{+} cells in *Tle4*$^{+/LacZ}$ and *Tle4*$^{LacZ/LacZ}$ cortices. **C**. FOXP2 staining, quantification by bin and the total numbers of FOXP2^{+} cells in *Tle4*$^{+/+}$ and *Tle4*$^{LacZ/LacZ}$ cortices. n=3 brains per genotype, 3 sections per brain. In all graphs, error bars represent ± SEM. Scale bars: low mag, 500 μm, high mag 100 μm.

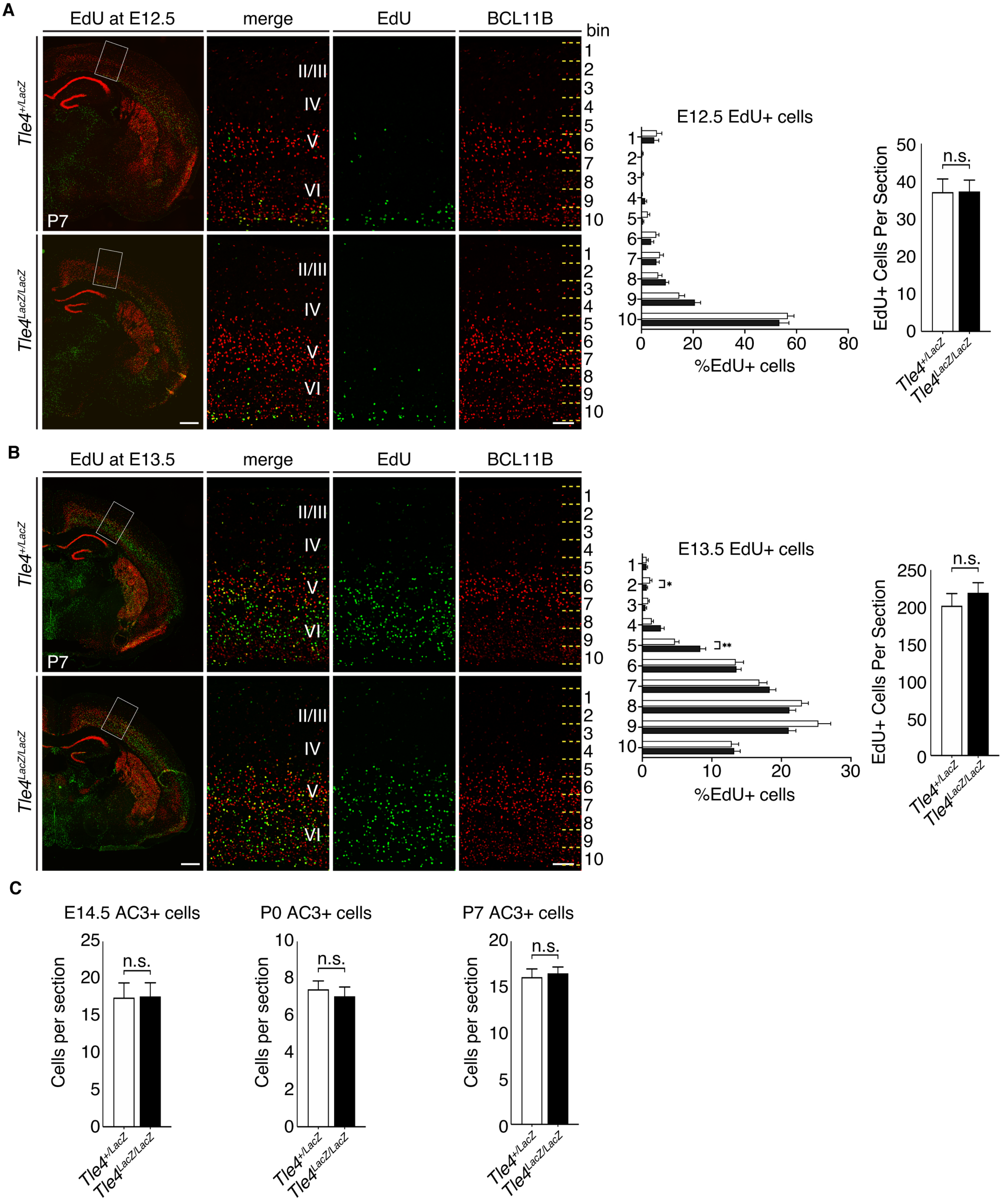

A
EdU at E12.5
merge
EdU
BCL11B
bin
Tle4+/LacZ
Tle4LacZ/LacZ
II/III
IV
V
VI
P7
E12.5 EdU+ cells
%EdU+ cells
EdU+ Cells Per Section
n.s.
B
EdU at E13.5
E13.5 EdU+ cells
C
E14.5 AC3+ cells
P0 AC3+ cells
P7 AC3+ cells
Cells per section

Figure S5. EdU birthdating and apoptosis analysis of the *Tle4*$^{+/LacZ}$ and *Tle4*$^{LacZ/LacZ}$ cortices.

Related to Figure 5.

A. EdU was given at E12.5, and brains were analyzed at P7. Quantifications show the %EdU^{+} cells per bin, and EdU^{+} cells per 750 μm wide section. **B**. EdU was given at E13.5, brains were analyzed at P7. Quantifications show the %EdU^{+} cells per bin, and total numbers of EdU^{+} cells per 500 μm wide section. **C**. Quantifications of AC3^{+} cells per section at E14.5, P0 and P7. n=3 mice per genotype and 3 sections per brain. Statistical significance for quantifications in **A-C** were determined using the unpaired student's t-test (*p<0.05, **p<0.01). Scale bars: low mag, 500 μm, high mag 100 μm.

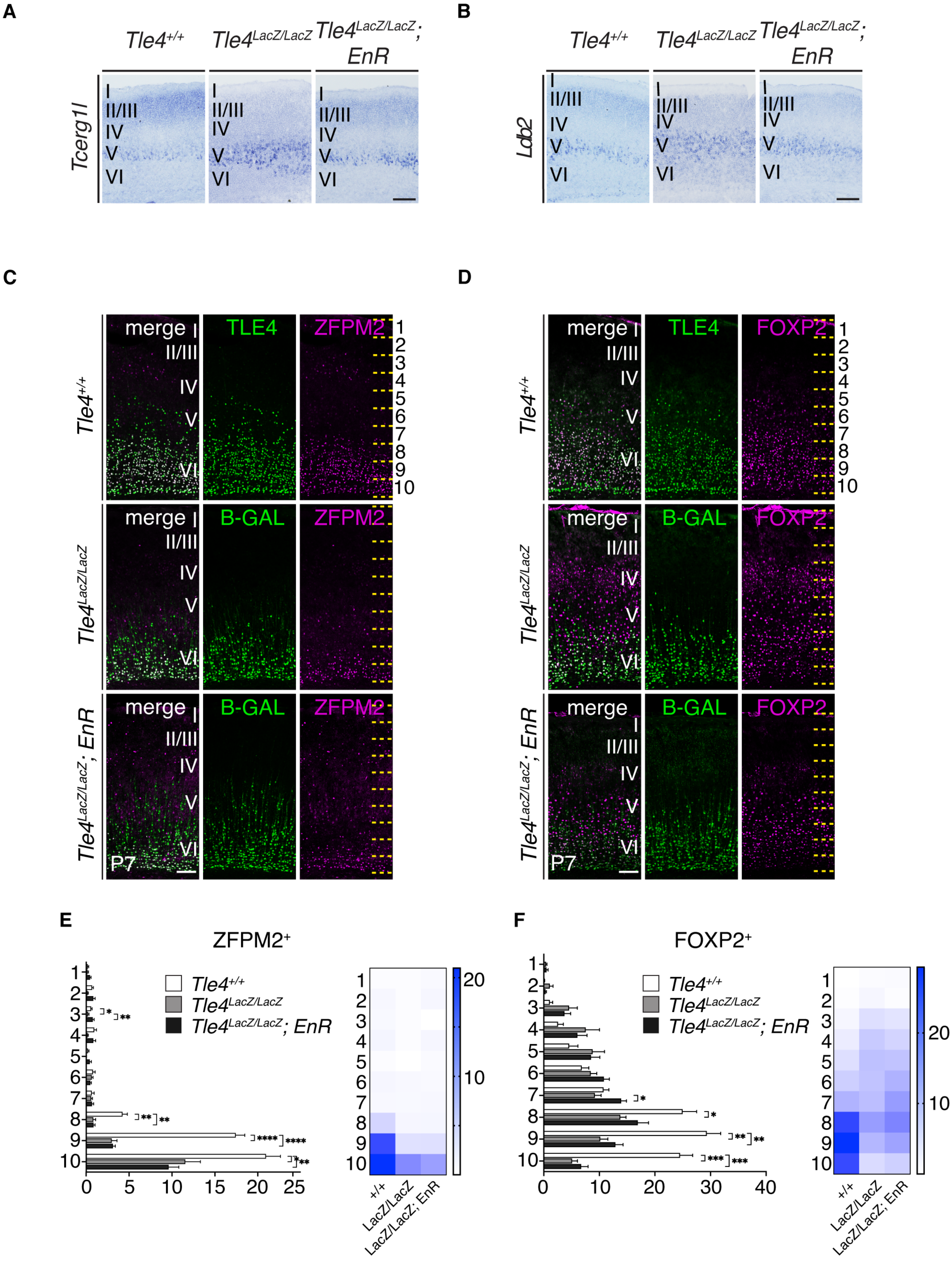

A
Tle4+/+
Tle4LacZ/LacZ
Tle4LacZ/LacZ; EnR
Tcerg1l
I
II/III
IV
V
VI
B
Tle4+/+
Tle4LacZ/LacZ
Tle4LacZ/LacZ; EnR
Ldb2
I
II/III
IV
V
VI
C
Tle4+/+
Tle4LacZ/LacZ
Tle4LacZ/LacZ; EnR
merge
TLE4
ZFPM2
B-GAL
I
II/III
IV
V
VI
P7
1
2
3
4
5
6
7
8
9
10
D
Tle4+/+
Tle4LacZ/LacZ
Tle4LacZ/LacZ; EnR
merge
TLE4
FOXP2
B-GAL
I
II/III
IV
V
VI
P7
1
2
3
4
5
6
7
8
9
10
E
ZFPM2+
Tle4+/+
Tle4LacZ/LacZ
Tle4LacZ/LacZ; EnR
0
5
10
15
20
25
20
10
+/+
LacZ/LacZ
LacZ/LacZ; EnR
F
FOXP2+
Tle4+/+
Tle4LacZ/LacZ
Tle4LacZ/LacZ; EnR
0
10
20
30
40
20
10
+/+
LacZ/LacZ
LacZ/LacZ; EnR

Figure S6. Increased expression of layer 5 subcerebral neuronal markers *Tcerg1l* and *Ldb2* were rescued, but reduced expression of layer 6 neuronal markers ZFPM2 and FOXP2 were not rescued in the *Tle4*$^{LacZ/LacZ}$ mice by the *Fezf2-EnR* allele.

Related to Figure 7.

A-B. In situ hybridization showed increased expressions for *Tcerg1l* and *Ldb2* in the *Tle4*$^{LacZ/LacZ}$ cortices, which were rescued in the *Tle4*$^{LacZ/LacZ}$; *Fezf2-EnR* mice. Scale bars: 100 µm. **C**. Immunostaining for TLE4, B-GAL, and ZFPM2 in the cortices of P7 *Tle4*$^{+/+}$, *Tle4*$^{LacZ/lacZ}$ and *Tle4*$^{LacZ/LacZ}$; *Fezf2-EnR* mice. Scale bars: 100 µm. **D**. Immunostaining for TLE4, B-GAL and FOXP2. Scale bars: 100 µm. **E**. Quantifications of the ZFPM2^{+} cells by bin. **F**. Quantifications of the FOXP2^{+} cells by bin. n=3 brains per genotype, 3 sections per brain. Quantifications of marker^{+} cells per 10,000 µm^{2} in each bin. In all graphs, error bars represent ± SEM. Statistical significance was determined using the unpaired student's t-test (*p<0.05, **p<0.01, ***p<0.001).
